# Machine Learning for Neurotransmitter Monitoring by Fast Voltammetry: Current and Future Prospects

**DOI:** 10.1021/acschemneuro.5c00543

**Published:** 2025-12-10

**Authors:** Cameron S. Movassaghi, Anne M. Andrews

**Affiliations:** † Department of Chemistry & Biochemistry, University of California, Los Angeles, Los Angeles, California 90095, United States; ‡ California NanoSystems Institute, University of California, Los Angeles, Los Angeles, California 90095, United States; § Department of Psychiatry and Biobehavioral Sciences, Semel Institute for Neuroscience and Human Behavior, and Hatos Center for Neuropharmacology, University of California, Los Angeles, Los Angeles, California 90095, United States

**Keywords:** electrochemistry, neurochemicals, multivariate analysis, chemometrics

## Abstract

Chemical neuroscience wields tools to uncover the molecular mysteries of the brain. Sensors can be fabricated with properties tailored to the scales needed to decode neurochemical information. Current instrumentation is capable of measurement rates that exceed neurochemical release rates. Modern machine learning models are approaching parameterization near the number of brain synapses. Fast voltammetry has remained a neuroanalytical workhorse technique for nearly half a century and has undergone significant transformations in many aspects due to advances in hardware and computation. Here, we review current and future uses of machine learning coupled with fast voltammetry to quantify neurochemical dynamics in the brains of behaving animal and human subjects. We focus on the advances that machine learning offers to pervasive problems in fast voltammetry. We identify current challenges and limitations for in vivo studies and delineate several routes for future development.

## Graphical abstract

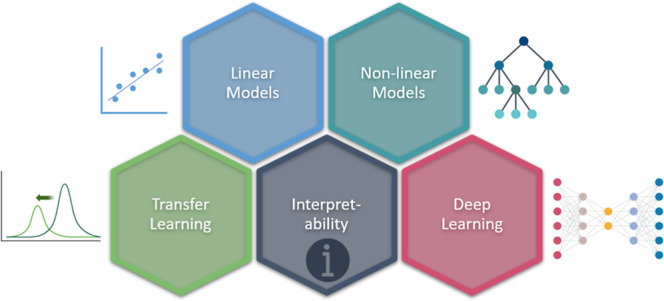

## 1 Introduction

Machine learning is poised to revolutionize the electrochemical sciences.^1^ The allure of machine learning lies in its ability to automate quantitative predictions from complex data sets across various data domains. Some of the most complex data sets and domains derive from brain chemical dynamics. To study brain molecular messengers at their relevant temporal, spatial, and chemical resolutions, a group of electroanalytical techniques encompassed by fast voltammetry has emerged to identify and quantify neurochemicals.^2–6^ Fast voltammetry utilizes waveforms that combine scan rates at hundreds of V/s and pulse sequences as short as milliseconds. Nonetheless, several key challenges in fast voltammetry limit its full potential for exploring molecular diversity in the brain and unlocking the chemical connectome.^7,8^

This Account discusses the challenges of performing brain fast voltammetry measurements and explores various data analysis approaches that aim to address current limitations. Slow-scan, nonvoltammetric, or nonbrain recording techniques are only mentioned briefly. Instead, our discussions are tailored toward in vivo neurochemical monitoring in the brains of behaving subjects (mainly mice, rats, and humans). The key benefit of the techniques covered is their ability to interpret real-time neurochemical dynamics in the context of behavioral processes.^9,10^ Molecular-scale knowledge of chemical neurotransmission will advance our understanding of the brain, from its normal circuitry to various pathologies and their treatments.^7^

Recent reviews on fast neurochemical voltammetry focus on specific neurotransmitters,^11^ electrode materials,^12–14^ and waveforms.^13,15^ Broader topics, such as in vivo monitoring techniques,^10,16^ machine learning in fundamental electrochemistry,^17,18^ or various types of electrochemical/biosensors, have also been reviewed.^19–22^ While machine learning has impacted experimental designs, analyses, and interpretations in neurochemical voltammetry, there are no dedicated reviews on this topic that we are aware of at the time of writing. Thus, we analyzed how machine learning is shaping the future of fast voltammetry, one of the most widely utilized methods for studying brain chemicals in real time. We identify trends and gaps in the literature and forward-looking paths for neuroscientists and electrochemists to draw inspiration. We draw parallels in analogous fields undergoing complementary development aided by machine learning, such as chemometrics, spectroscopy, and electrochemistry *writ large*. Thus, this review will also be of interest to domain experts in these areas.

### 1.1 Why Detect Neurotransmitters Using Fast Voltammetry?

Compared to alternative techniques (e.g., microdialysis, genetically encoded sensors, and biosensors), fast voltammetry offers an attractive combination of physiologically relevant sensitivity and spatiotemporal resolution.^10^ Figures of merit can be optimized by adjusting waveform parameters, electrode materials, coating choices, and data analysis procedures (Figure 1).^11,15,23–27^ Nevertheless, a single technique for detecting multiple neurochemicals in the brain across various recording locations, time scales, and behavioral states remains elusive.^7,8,28–30^ Fast voltammetry is limited to electroactive analytes and by continuous measurement duration (i.e., recording time scales of seconds to minutes rather than hours to days). There are also challenges in chemical selectivity and simultaneous multianalyte detection (multiplexing) and problems with calibrating electrodes in vitro for in vivo studies (generalizability). Given the growing amounts of data collected, the need to quantify in complex deployment environments, and an expanding panel of neurochemicals, materials, and waveforms, machine learning is poised to unlock the full power of fast voltammetry (Figure 1).

**Figure 1:**
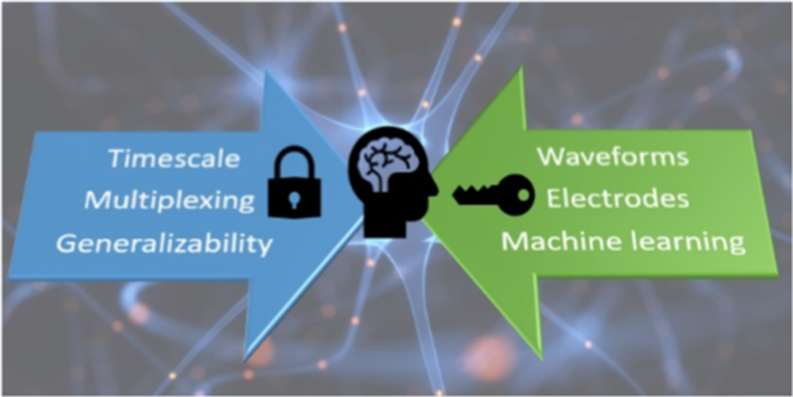
Grand challenges and solutions in fast voltammetry.

### 1.2 What Is Machine Learning, and Why Is It Used?

Machine learning is when a “*computer program is said to learn from experience E with respect to some class of tasks T, and performance measure P, if its performance at tasks in T, as measured by P, improves with experience E*”.^31^ Analogous terms, including statistical learning, deep learning, artificial intelligence, and chemometrics, are sometimes used interchangeably with machine learning. Full treatments of the machine learning models and algorithms discussed herein are referenced as needed.

Machine learning is broadly classified into unsupervised, which utilizes unlabeled data, supervised (labeled data), semisupervised (labeled and unlabeled data), and reinforcement (feedback loop) learning (Figure 2). We focus on supervised learning because the task is to predict unknown concentrations in vivo from known concentration samples used for in vitro training data for a given panel of neurochemicals. We briefly mention approaches for classification (i.e., whether a voltammogram contains dopamine or not) but maintain that the most powerful applications of machine learning and fast voltammetry in terms of discovering new biology will result from the exploration of quantitative changes in multiple neurotransmitters, rather than simply identifying their qualitative presence.

**Figure 2:**
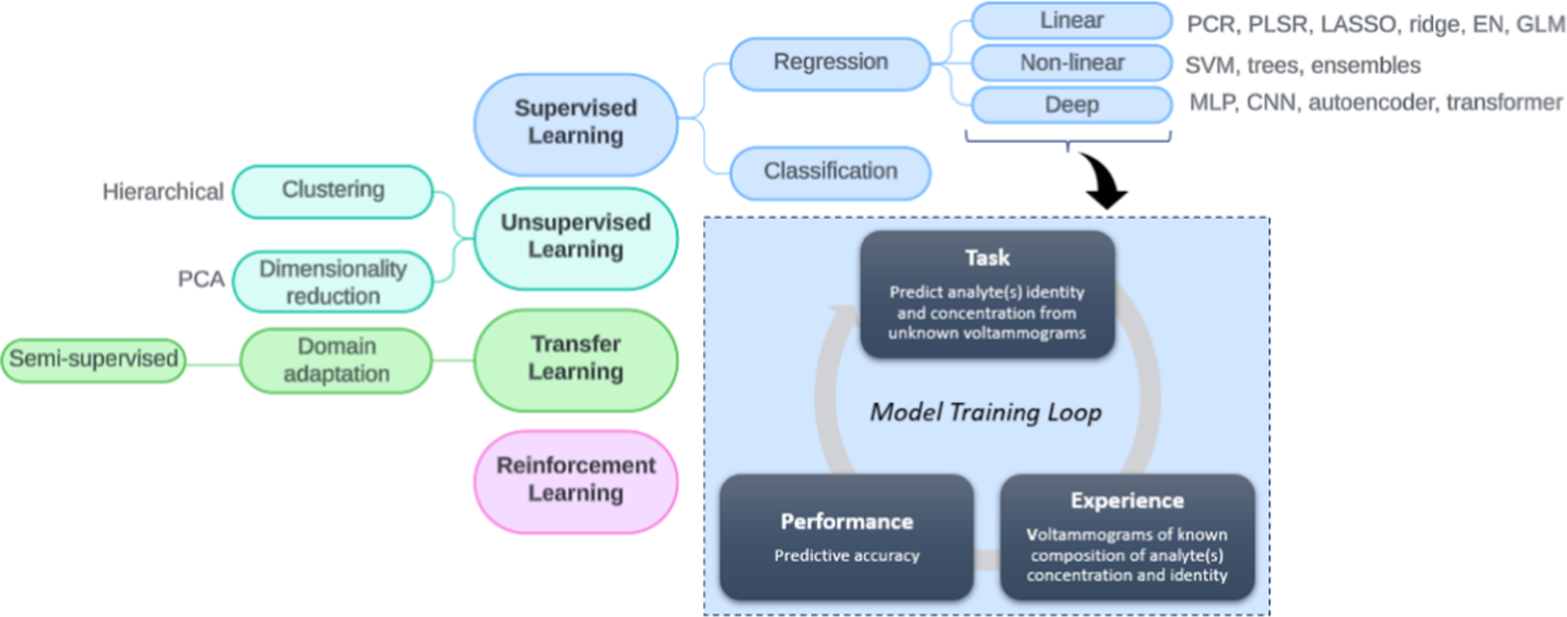
Approaches to machine learning in fast voltammetry, including principal components analysis (PCA), principal components regression (PCR), partial least-squares regression (PLSR), least absolute shrinkage and selection operator (LASSO), elastic net (EN), generalized linear model (GLM), support vector machine (SVM), multilayer perceptron (MLP), and convolutional neural network (CNN).

Some of the earliest research on voltammetry and machine learning dates back to the 1970s, when classification and/or clustering approaches were used to deconvolute overlapping polarograms.^32,33^ Since the advent of computers for controlling chemical instrumentation, machine learning has been pursued to enhance the resolving power of voltammetry signals. Before the use of machine learning, conventional voltammetric discrimination of analytes in aqueous (i.e., physiological) solution was thought to be limited to <15 compounds (even under ideal conditions and generous solvent windows).^34^ Monitoring more than one neurotransmitter continuously using voltammetry was a noteworthy feat. Today, monitoring multianalyte mixtures is becoming increasingly common, offering unparalleled accuracy, sensitivity, and selectivity.^26,35–37^

### 1.3 Structuring Voltammetry Training Data for Machine Learning

Voltammetry input (training) data **X** can be structured in a tabular format with design matrices *N* × *D,* where *N* is a single or averaged voltammogram (row) and *D* is a feature (e.g., sampled data point within a voltammogram) (Figure 3). In fast voltammetry, data points are collected across voltammograms at hundreds of kHz up to ∼2 MHz sampling frequencies; *D* > 1000. This sampling is repeated for each voltammogram (waveform cycle), with voltammograms typically collected at a rate of 10 Hz. Historically, when a small number (e.g., dozens) of voltammograms were used to build a training set, this resulted in *D* ≫ *N* (e.g., “wide” training data rather than “big” training data^38^). Modern fast voltammetry training sets are transitioning into the “big” regime, where the number of training voltammograms (e.g., tens of thousands or more) exceeds the number of sampled points in a voltammogram.

**Figure 3:**
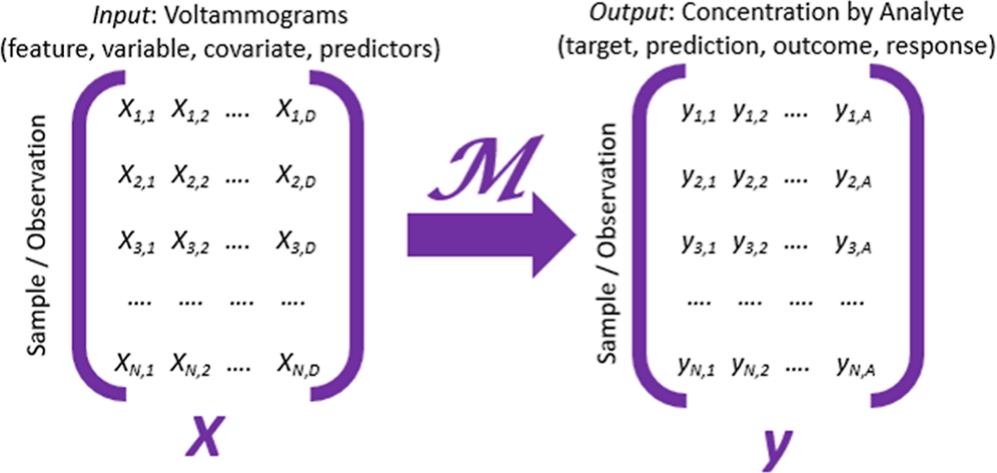
Format of vectorized voltammetry data as input (*X*) and output (*y*) to train on or predict from a model (*M*).

In the simplest case, training data are obtained using a single electrode in a single experiment, and inference, i.e., prediction of concentrations from in vivo experimental data, is performed for experimental (in vivo) data collected using the same electrode (i.e., “within electrode”). However, as discussed below, data from multiple electrodes can be combined, and inference may or may not be performed on data from the same electrode (“across electrodes” or “out-of-probe”; Section 5.1). The model used to perform inference (*M*) is trained on and outputs predictions as a matrix ***y*** of shape *N* × *A*, where *A* is the number of analytes (i.e., for one analyte, only one column is output). Each row (*N*) in ***y*** corresponds to the known or predicted analyte(s) and concentration(s) of each voltammogram in ***X***. When the voltammograms in ***X*** are ordered sequentially across time, the resulting predictions in ***y*** yield plots of analyte concentrations vs. time, which are used for visualizing in vivo dynamics and correlating analyte concentration changes with known stimuli.

Within-electrode data are obtained using a single working electrode. Multi-electrode models are trained using data from multiple electrodes. If predictions are made on an electrode(s) not included in the training set, the multi-electrode model is performing “out-of-electrode”.

### 1.4 Information Contained within Voltammetry Data

Voltammetry data consist of four main experimental variables: the voltage sequence applied at an electrode surface (the waveform), the resulting measured electrode current (the voltammogram), the data point sampling time (sampling frequency), and the time between waveform cycles (waveform frequency). Voltammograms provide information about the physicochemical properties of an analyte, including concentration, diffusion, adsorption, electron transfer kinetics, impedance, and waveform-generated capacitance. Fundamental electrochemical theory and formulas are available to describe these relationships.^39^ Moreover, research is being done to parameterize these relationships and incorporate electrochemistry and physics into machine learning models.^40–42^

Before the use of machine learning, analyses of voltammograms involved identifying characteristic peak positions and shapes. However, pattern matching or extending known theory can be difficult at high scan rates and under nonideal experimental conditions encountered in fast voltammetry. As an alternative, automated analysis approaches have been employed to identify neurochemical voltammogram patterns for analytes such as adenosine and dopamine.^43–46^ These are rules-based approaches rather than models with a range of learnable parameters. Instead, machine learning in fast voltammetry focuses on modeling empirical relationships inherent in the evoked current as a function of the quantities of analyte(s) present. These relationships are learned from the standard training data.

### 1.5 Approaches to Model Training, Validation, and Testing

#### 1.5.1 Training Sets

Neurochemical voltammetry seeks to fit a model empirically to the relationship between signal response at an electrode (voltammogram for an applied waveform) and the electrode environment (identity and concentration of analyte(s)). Iteratively updating the parameters of the underlying calibration model is referred to as training, and the data set used for parameter updating is known as the training or calibration set. Ideally, training sets should contain all expected sources of variation, scale with the degrees of freedom of the model, span all concentration ranges and mixtures of analytes/interferents expected to be encountered in vivo, and be collected under the same conditions and with the same equipment (e.g., sources of noise) as the test set, defined below.^47^

Training set data can be acquired in vitro or in vivo. We discuss in vivo training sets in detail in Section 5.3. In vitro training sets are increasingly common and are acquired by using a flow cell with sequential injections of standards (Figure 4). For these standards, neurochemicals of interest are prepared at known concentrations in a physiological buffer (e.g., artificial cerebrospinal fluid). Training sets can be as simple as, for example, five concentrations of dopamine or as complex as several neurochemicals, each across dozens of concentrations, mixtures, and noise conditions. Training set standards are injected at controlled intervals, while physiological buffer flows continuously through the flow cell. Training voltammograms are extracted from a stable response area during each injection such that the electrode is fully exposed to the sample. To a rough approximation, this process mimics the rapid release and reuptake of neurochemicals in a reproducible and straightforward manner. Efforts are underway to improve flow cell design using microfluidics and 3D printing.^48,49^

**Figure 4:**
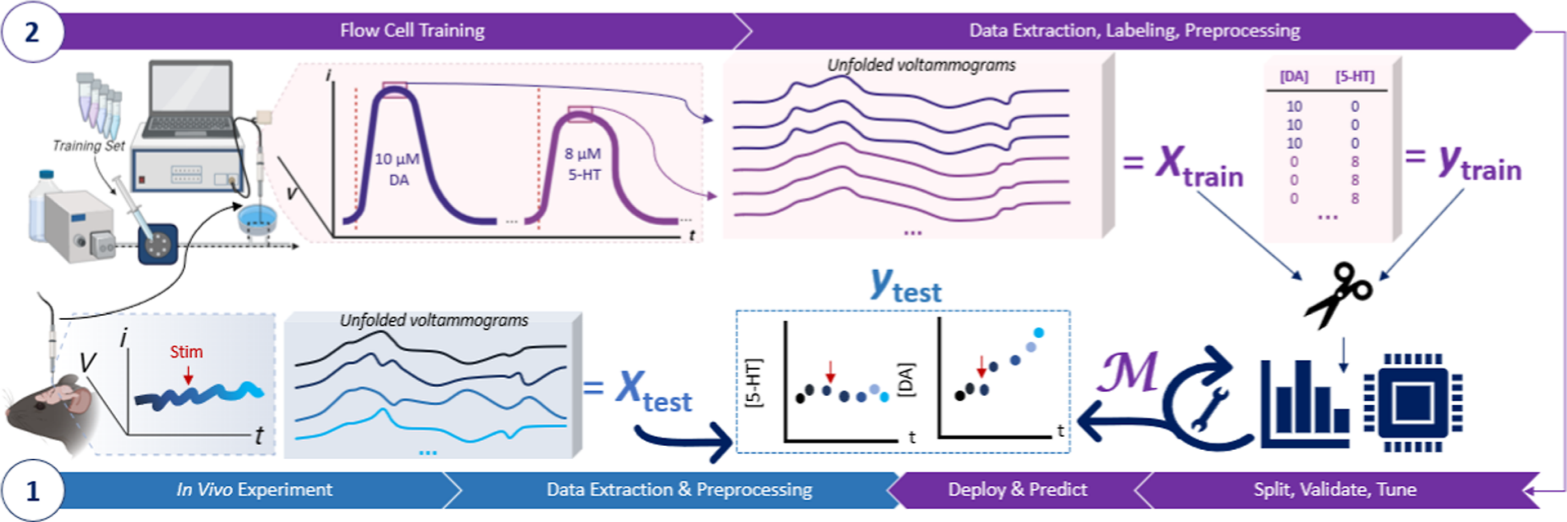
Use of flow-cell sequential injection analysis for electrode calibration, model training, and inference. An electrode is generally first used in vivo (1), followed by postfouling calibration (2), also known as postcalibration. “Stim” refers to a stimulation (e.g., pharmacological, optical, electrical, or behavioral). Representative analyte calibration data are obtained (e.g., dopamine (DA), 5-hydroxytryptamine/serotonin (5-HT), etc.) using a flow cell. The data are split, and the model is trained, tuned, and validated before the final model *M* is used to perform inference on (predict) the unknown in vivo data (i.e., test data).

Anywhere from one to hundreds of voltammograms may be extracted from a single injection, each corresponding to a standard’s known concentration. All voltammograms from a single standard can be averaged or treated as replicates. The final size of the data set depends on the number of injections, the number of voltammograms extracted per injection, the sampling and waveform frequencies, and possible preprocessing (described below). Outlier removal procedures (e.g., Cook’s distance) can be utilized to remove poor training data.^50^ With a data set in hand, the training, validation, and testing of a suitable machine learning model are performed by dividing the data accordingly.

Neurochemical voltammetry seeks to fit a model, empirically, to the relationship between signal response at an electrode (voltammogram for an applied waveform) and the electrode environment (identity­(ies) and concentration(s) of analyte(s)).

#### 1.5.2 Validation and Test Sets

We distinguish between two uses of the term “validation”. In neurochemical voltammetry, validation refers to the process of testing a new voltammetry technique under neurobiological challenges (e.g., ex vivo or in vivo) with known effects. These challenges can include pharmacological, stereotaxic, or stimulation-based (i.e., optical, electrical, and behavioral) experiments in which the expected responses of certain neurochemicals of interest are known (e.g., optogenetic stimulation of dopaminergic neurons should produce a dopamine response).^26^ If the results align with domain knowledge, then the technique is considered validated.

In machine learning, model validation refers to holding out a portion of the training data, known as the validation set, to estimate the model performance on unseen data (i.e., cross-validation). This is done such that reasonable hyperparameters can be chosen before deploying the fully trained model with an unknown test set. The validation set comprises known but unlabeled input data that the model has not encountered during its construction. Validation set data are used to gauge model generalizability and to reduce model overfitting. Once a model is trained, it can be used to predict neurotransmitter concentrations sequentially from in vivo recordings. These continuous temporal plots, spanning seconds to minutes, enable the construction of a biological time course with known experimental events at defined timestamps.

Model training can be carried out using a training set before in vivo experiments, referred to as precalibration in conventional experiments. Next, in vivo experimental data are collected at the implanted electrode. Postcalibration training can also be performed by removing the electrode and using it to obtain additional in vitro training data. Model training after implantation and biofouling, and across multiple (similar) electrodes for large, chemically diverse analyte panels, provides data more similar to the test data, as electrodes have been exposed to brain tissue, which can alter electrode surfaces and the resulting calibration responses.^5,51,52^ An appropriately trained model should retain only the salient, conserved, latent voltammetric features across these environments to generalize.

## 2 Linear Methods

Notably, all in vivo neurochemical concentrations rely on model predictions. Fast voltammetry calibration has historically used univariate linear regression to predict concentrations of neurochemicals from voltammograms. Peak currents at characteristic voltages (e.g., the peak dopamine oxidation potential) are plotted as functions of known analyte concentrations, allowing the concentration of an unknown (in vivo) voltammogram to be predicted.^53^ Because peak amplitudes (or areas under the curve^46^) at characteristic potentials are proportional to analyte concentrations,^39^ linear models were a first choice for fast voltammetry. Other uses of linear regression in fast voltammetry include correlation analyses of voltammogram “templates”,^5,43,54^ and setting integration boundaries for calculating charge under a faradaic peak.^55^

However, univariate linear regression neglects the majority of the data in the voltammograms. It suffers drawbacks associated with voltage-peak overlap for species common in brain voltammetry measurements (e.g., dopamine and pH oxidation peaks overlap and both analytes change simultaneously).^56^ An attractive alternative is multivariate linear regression, which reduces noise and interferent effects (Figure 5). (Note that nonbackground-subtracted data are shown to illustrate possible covariates. Historically, background subtraction is performed prior to regression. An in-depth review on the principles of faradaic and nonfaradaic current, and the use of background subtraction in fast voltammetry can be found elsewhere.^4^) However, voltammograms contain multicollinear data (i.e., multiple points within a voltammogram are correlated with the same analyte), and data sets with more features than samples (*D* ≫ *N*) result in regression problems with nonunique or unstable solutions.^47,57^ Thus, methods for selecting which variables in the voltammograms to use as predictors are often required.

**Figure 5:**
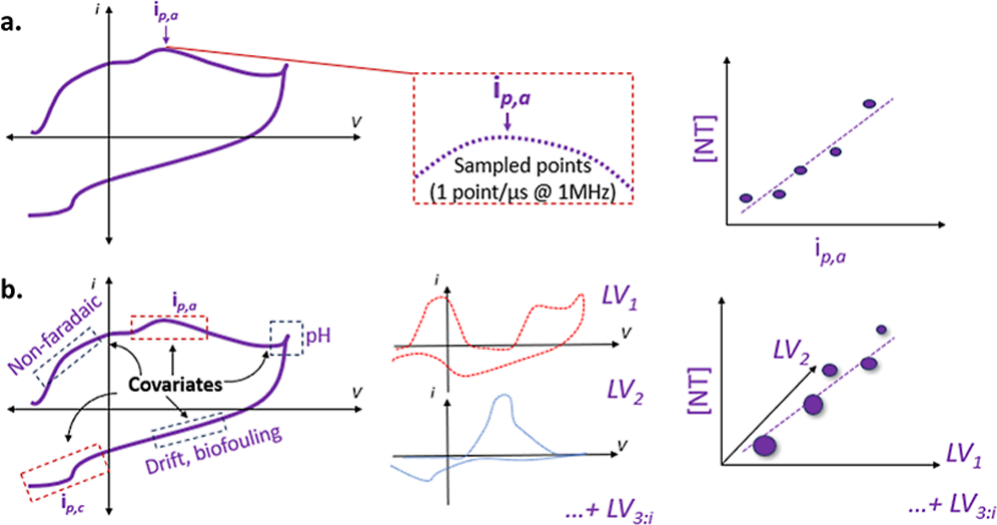
(a) Univariate linear regression as typically carried out in fast voltammetry, where NT = neurotransmitter. (b) Multivariate linear regression with latent variables. Voltammograms are nonbackground subtracted.

### 2.1 Dimensionality Reduction

One approach to dealing with multicollinearity is dimensionality reduction. Dimensionality reduction creates linear combinations of predictors that explain the variance in a data set.^47^ One of the earliest applications of machine learning for fast voltammetry of neurochemicals was PCA followed by linear regression, known as PCR.^34^ The PCA technique is considered an unsupervised method because the correlation between the input ***X*** data and the concentration data (***y*** matrix) is not considered; only the variance in ***X*** itself is considered. When the concentrations are regressed on the principal components using linear regression, model fitting and predictions of concentration levels for each analyte in the training set can be made. In-depth discussions and tutorials for PCA and PCR in fast voltammetry can be found elsewhere.^34,50,58–61^

The first application of PCR to fast-scan cyclic voltammetry (FSCV-PCR) of neurochemicals reduced the data set from a 1000-dimensional space per voltammogram to 5 dimensions (i.e., a data set of 30,000 data points was reduced to 150 data points). In addition, the selectivity was improved by separating the variance contributions in the data (Figure 6). Ascorbate, serotonin, 3,4-dihydroxyphenylacetic acid (DOPAC), and pH were resolved from dopamine, respectively, in two-component mixtures within 10% accuracy for dopamine. The PCR method was validated in rat striatum brain slices, where it differentiated dopamine release (hundreds of nM) vs. a pH change of >0.1 units after electrical stimulation. The same study further validated the use of PCR using bovine adrenal medullary cells and revealed vesicular release of norepinephrine and epinephrine. While PCR could discriminate the latter analytes, the temporal resolution of voltammetry was limited as sufficient time was required for epinephrine to adsorb and produce the unique secondary oxidative wave that differentiates it from norepinephrine.

**Figure 6:**
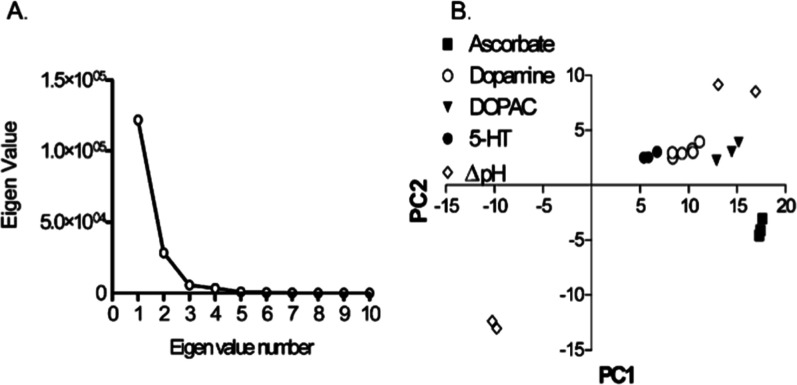
Early application of PCR to fast-scan cyclic voltammetry data for neurochemical prediction. DOPAC = 3,4-dihydroxyphenylacetic acid, 5-HT = 5-hydroxytryptamine, and serotonin. Reprinted with permission from Heien, ML; Johnson, MA; Wightman, RM. *Anal. Chem* **2004** 76 (19), 5697–5704. Copyright 2004 American Chemical Society.

A follow-up study validated PCR for quantifying dopamine and pH changes in the rat nucleus accumbens.^62^ Here, an in vivo training set was used to address the shortcomings of in vitro training sets, discussed fully in Section 5. Briefly, a major reason for in vivo, as opposed to in vitro calibration, is matching the domains of training vs test set data. For example, the voltammogram response to pH changes,^56^ sources of noise, electrode fouling, and reference electrode offsets^63^ are substantially different in vivo compared to in vitro. By using in vivo training data to predict in vivo test data, techniques that assume matched domains (such as Q_α_, which assumes that training set noise mimics test noise) are more reliable.^50^ The in vivo training set was acquired by varying the electrical stimulation pulse and frequency settings as voltammograms were acquired in the rat brain (see also Section 5.3).

Since these two landmark studies in the early 2000s, PCR combined with a triangle FSCV waveform for dopamine detection remains one of the most commonly utilized approaches in the field.^64,65^ Extensions and modifications of the FSCV-PCR approach followed. For example, including background drift in the training set enabled the separation of drift contributions from dopamine and pH.^66^ Co-detection of glutamate and dopamine using an enzyme-modified electrode and a modified FSCV triangle waveform, respectively, was reported using PCR.^67^ Other analytes detected using FSCV-PCR have included adenosine,^68^ oxygen,^69^ and serotonin and dopamine,^70^ as well as adenosine triphosphate and hydrogen peroxide using a sawhorse waveform,^71^ and a dual-waveform for detecting norepinephrine and dopamine.^72^ Waveform modifications for lowering the detection limit for PCR have also been studied.^73^ As an alternative to PCR, multivariate curve resolution with alternating least-squares was applied to FSCV data, yielding results that performed similarly to PCR for dopamine and pH predictions, albeit with more complex analyses.^74^

A technique related to PCR is PLSR. The origin of the PLSR approach is rooted in chemometrics. Compared to PCR, PLSR is considered a supervised dimensionality reduction approach.^75^ While PCR models the variance in ***X*** with the assumption that it relates to ***y***, PLSR explicitly considers the covariance of ***X*** and ***y*** when building components, resulting in a higher weighting of input variables that correlate with responses. Thus, PLSR creates dimensions of voltammogram features that relate to concentration and current, rather than solely to current variations (which could include noise), often resulting in fewer retained components in PCR to explain a data set.

In contrast to FSCV, we have used a rapid pulse waveform and the PLSR approach to codetect dopamine and serotonin in mouse striatum.^26^ A recent report used a modified FSCV waveform with PLSR to detect serotonin and histamine in human hair follicle epithelial cells.^76^ The Sombers group reported several PLSR-based techniques with a double waveform for drift subtraction and pH/H_2_O_2_ differentiation.^27,77^ The PLSR approach outperforms PCR for predicting neurochemical concentrations, as shown for fast and slow-scan waveforms and rapid pulses.^26,78,79^ However, PCR remains the more common approach, perhaps due to its historical use and the abundance of fast voltammetry-specific PCR tutorials.^59,60^ The PCR approach was also implemented in several software packages for FSCV,^80,81^ while other analyses require offline coding knowledge. Many extensions of PCR and PLSR exist, including nonlinear adaptations; however, few have been explored for FSCV. For example, Loewinger reported on the use of functional PCR (fPCR) but found it had inferior performance to PCR.^82^

The introduction of PCR to fast voltammetry led to a series of papers on best practices for training and tuning accurate predictive machine learning models in vitro or in vivo, to translate them for in vivo use. Discussions of best practices are ongoing today. The two examples below focus on selecting components and validating them through residual analysis. The use of multiple electrodes and the construction of valid training sets are discussed in the context of generalizability (Section 5).

The inaccessibility of in vivo ground-truth is the root cause of disagreement about the use of machine learning for neurochemical voltammetry.

#### 2.1.1 Choosing Components

Choosing the number of components is the main hyperparameter tuned in PCR and PLSR models. A hyperparameter is a model-specific variable that is set before training as opposed to a parameter that is learned during the training process. The components found most valuable for describing the data and performing predictions are usually termed primary or retained components. In contrast, the discarded components are referred to as secondary or noise components.^47^ Hyperparameter tuning helps address the bias-variance trade-off. If the threshold is set too low and too few components are retained, the data are underfitted, and useful information is discarded. If too many components are retained, then the data are overfitted by modeling noise.

While numerous approaches and discussions on selecting the number of components exist in chemometrics, three primary approaches are most commonly utilized for neurochemical fast voltammetry. These are cumulative variance threshold, Malinowski’s F-test, and cross-validation. Setting the number of components based solely on a priori knowledge of the sources of variation is strictly not recommended (e.g., a two-component model for a training set for pH and dopamine), as components may or may not be physicochemically relevant to the analytes (and alternative methods exist if this is desired).^74,83^

Cumulative variance was employed in the seminal work on FSCV-PCR, and this approach remains in use today due to its simplicity.^34^ Here, all ordered components explaining up to 99.5% of the variance are designated as primary components, while the remaining components are defined as secondary components and discarded. However, this method assumes that noise makes up an arbitrary percentage (e.g., 0.5%) of each data set and is thus unlikely to generalize across experiments, electrodes, or laboratories.

A formal technique grounded in chemometrics is Malinowski’s F-test. This technique sets a data set-dependent threshold of statistical significance α (e.g., 5–10%) to delineate primary and secondary component assignments. The F-test was compared to cumulative variance selection for PCR data.^58^ Here, 119 in vivo training sets, each containing five dopamine and five pH voltammograms, were analyzed across different technicians, laboratories, and electrodes. Of the 119 training sets, only 25% resulted in the same choice of components by cumulative variance vs F-test, with cumulative variance often retaining more components than the F-test. The F-test removed significantly more noise by selecting an appropriate number of components compared to cumulative thresholds for dopamine and pH data. However, predictions remained virtually unchanged in certain cases, even when up to four additional secondary components were retained (i.e., predictions made by models with components set by either method were highly correlated). A follow-up study demonstrated that outlier removal techniques, such as Cook’s distance, can be used to guide rank selection.^50^

Cross-validation can also be used to select the number of components. Here, a portion of the training set is withheld to function as a validation set. Models are trained with the remaining training data. A model is then used to make predictions for the validation set as the number of components in the model varies. Cross-validation has been compared to the F-test method with mixed results, as determining the optimal number of components by cross-validation can be ambiguous (e.g., determining the “elbow” point in a Scree plot of variance explained as a function of retained components), while the F-test relies on strict statistical assumptions.^84^

One recent report found that cross-validation outperformed the F-test, specifically for FSCV PCR data.^85^ Regardless, cross-validation remains the de facto technique in the wider machine learning community, although various component selection methods continue to be explored.^84^ For fast voltammetry with PLSR, cross-validation remains the most popular method for component selection. Notably, the F-test for PLSR component selection has seen little to no use in fast voltammetry, perhaps because PLSR is a supervised technique. A failure of all component-based techniques is that relevant information can be contained within noisy, secondary components.

Many types of cross-validation exist. When *k*-fold cross-validation is used, a certain percentage (defined as a “fold”) of the data is held out. For example, 5- and 10-fold cross-validation are common in voltammetry, where 20% or 10% of the data (e.g., 5 or 10-fold) is randomly withheld. The folds can also be grouped or stratified by concentration or electrode, allowing for out-of-concentration or out-of-electrode cross-validation.^86^ Further, some techniques use a portion of the scans from the same flow cell injection to hold out, while others group solely by separate injections.^36^ Choosing components by cross-validation is becoming more commonplace (Table 1), as is done for most hyperparameter optimizations, as the computation involved in large iterations of training during cross-validation has become exceedingly fast. Nevertheless, the choice of components is a broader topic in the fields of chemometrics and machine learning, which has only recently been explored in fast voltammetry (Section 5.2).

**Table 1:** Representative Sample of Novel Applications of Machine Learning to Quantify Neurochemicals Using Fast Voltammetry^a^

year	reference	waveform/technique	model(s)	analyte(s) and interferents	model validation	training set	animal validation/application	# electrodes
2004	^34^	triangle	PCR	DA, 5HT, DOPAC, AA, pH, NE, EP, 3MT, l-DOPA, UA, HVA	VT (99.5%)	in vitro	rat striatum (slice); electrical stimulation; and vesicular release	1
2016	^85^	triangle	PCR, EN	DA, pH	10 fold CV, F-test	in vitro (out-of-probe)	human striatum; sequential investment game	≥2
2018	^88^	triangle	LASSO	5HT, DA, pH	10 fold CV	in vitro (out-of-probe)	human striatum; sequential investment game	3
2019	^79^	Triangle	PCR, PLSR	DA, AA, DOPAC, 5HT, pH	VT (99.9%), 10-fold CV	in vitro	n/a	3
2019	^92^	random burst	EN, LASSO	5HT, DA, pH, NE, 5HIAA	10 fold CV	in vitro	n/a	n/a
2020	^126^	triangle	PCR, PLSR, MLP	DA	10 fold CV, 5 components	in vitro	n/a	5
2020	^89^	triangle	EN	5HT, DA, pH	10 fold CV	in vitro (out-of-probe)	human striatum; visual motion discrimination task	20
2020	^115^	triangle	SVR	DA, 5HT, AA, DOPAC, pH	5 fold CV	in vitro	rat nucleus accumbens; electrical stimulation	n/a
2021	^127^	triangle	Autoencoder	AA, DA, NaCl	n/a	in vitro and in vivo (semisupervised)	rat striatum; pharmacological validation	1
2021	^26^	triangle, rapid pulse	PCR, PLSR	DA, 5HT	LOOCV	in vitro (postcalibration)	mouse striatum; stereotaxic, pharmacological, and optogenetic validation	3
2021	^111^	MCSWV	GLM	DA	5 fold CV	in vitro (postcalibration)	rat striatum; pharmacological validation	6
2021	^91^	low-amplitude burst, low-amplitude triangle	EN	5HT, DA, pH, EP	10 fold CV	in vitro	n/a	n/a
2022	^36^	triangle	PCR, CNN	DA, EP, NE, 5HT, pH	VT (99.99%), 5 fold CV	in vitro (postcalibration)	rat striatum; electrical and pharmacological validation	5
2022	^86^	triangle	MLP, CNN, transformers	DA, 5HT, NE, pH	10 fold CV	in vitro	n/a	76
2022	^130^	N-shape	MLP	5HT	10 fold CV	in vitro (postcalibration)	mouse hippocampus; electrical and pharmacological validation	140
2022	^131^	N-shape	CNN	5HT, DA	5 fold CV	in vivo (data augmentation)	mouse hippocampus; electrical and pharmacological validations	10
2023	^35^	triangle	CNN	NE, DA, 5HT, pH	10 fold CV	in vitro (postcalibration)	human amygdala, visual affective oddball task	6
2024	^37^	MCSWV	Autoencoder, SVR, LASSO, EN, Ridge, PLSR, PCR	DA, NE, 5HT, UA, pH, DOPAC, AA, ADO, HVA	5–20 fold CV, VT (99%)	in vitro and in vivo (semisupervised; out-of-probe)	rat nucleus accumbens, pharmacological validation	12

a 3MT = 3-methoxytyramine, 5-HIAA = 5-hydroxyindoleacetic acid, 5-HT = serotonin, AA = ascorbic acid, ADO = adenosine, CNN = convolutional neural network, CV = cross-validation, DA = dopamine, DOPAC = 3,4-dihydroxyphenylacetic acid, EN = elastic net, EP = epinephrine, GLM = generalized linear model, HVA = homovanillic acid, LASSO = least absolute shrinkage and selection operator, l-DOPA = levodopa, MCSWV = multiple cyclic square wave voltammetry, MLP = multilayer perceptron, n/a = not applicable/not reported/unclear, NE = norepinephrine, PCR = principal components regression, PLSR = partial least squares regression, SVR = support vector regression, UA = uric acid, and VT = variance threshold.

#### 2.1.2 Q_α_ Validation

A model validation technique developed specifically for in vivo fast voltammetry with PCR compares the residuals of reconstructed and raw voltammograms, referred to as the Q_α_ method.^62^ If the residuals between the reconstructed (via retained PCs) and raw voltammograms of the unknown data set (i.e., in vivo data) do not fall within a given threshold α (unrelated to α in the F-test, but also usually set to 5–10%), the data are rejected as inaccurately quantitated by the model (i.e., a significant source of variance is not accounted for by the training set but is present in the test set).^58^ In theory, this approach rejects voltammograms with unaccounted-for interferents, which are estimated by the model. This approach makes no guarantees on a model’s accuracy, only on its applicability.^59,60^ Some reports maintain that because Q_α_ values are distributed in a manner dependent on the data set, rather than a single value, a universal training set does not exist for in vivo training data.^58^ While the Q_α_ approach helps identify potentially erroneous voltammograms simply by way of input, noisy training sets can inflate Q_α_ values such that inappropriate voltammograms are retained.^61^ Furthermore, this method assumes that an accurate prediction (output) is correlated to an accurate signal reconstruction (input).

### 2.2 Regularization

Regularized linear regression has been explored as an alternative to PCR and PLSR for analyzing fast voltammetry data. Regularization prevents overfitting and addresses multicollinearity by adding a penalty term to the regression coefficients during training. In voltammetry, rather than defining new dimensions as combinations of predictors in PCR/PLSR, regularization treats each sampled point in the voltammogram as an independent predictor. Each predictor’s regression coefficient is penalized to shrink its value toward zero, thus reducing variance.

Regularized approaches include ridge, least absolute shrinkage and selection operator (LASSO), and EN regression.^87^ Each of these techniques differs in how it penalizes the regression coefficients. For example, ridge regression shrinks the 1_2_ norm of the regression coefficients, while LASSO utilizes the 1_1_ norm. A benefit of LASSO is its sparsity, which allows the regression coefficients to be exactly zero. The EN approach combines both the 1_1_ and 1_2_ penalties. None of the three approaches will outperform the others universally; they must be selected and tuned by using cross-validation.

Regularization approaches (referred to here as EN electrochemistry) were pioneered in fast voltammetry by the Kishida and Montague groups, who also developed working electrode procedures for in-human use.^6,65^ Accordingly, EN electrochemistry has been primarily used in conscious human subjects undergoing neurosurgical procedures (i.e., deep brain stimulator implantation). The first report was in 2016, where EN was used for dopamine prediction in 17 human subjects.^85^

A follow-up study investigated serotonin signaling in 14 humans using LASSO,^88^ and later, serotonin and dopamine codetection was performed in four humans again using EN (Figure 7).^89^ All studies were conducted in the striatum, while the participants performed decision-making tasks under uncertainty, such as a stock investment game. In vitro, the EN approach has been extended to the detection of oxytocin and vasopressin,^90^ as well as to low-amplitude sweep and random-burst pulse waveforms.^91,92^ EN electrochemistry is commonly used with large training sets across many electrodes, such that the coefficients of predictors with high variance shrink toward zero, effectively canceling out points in voltammograms that cause out-of-electrode effects.

**Figure 7:**
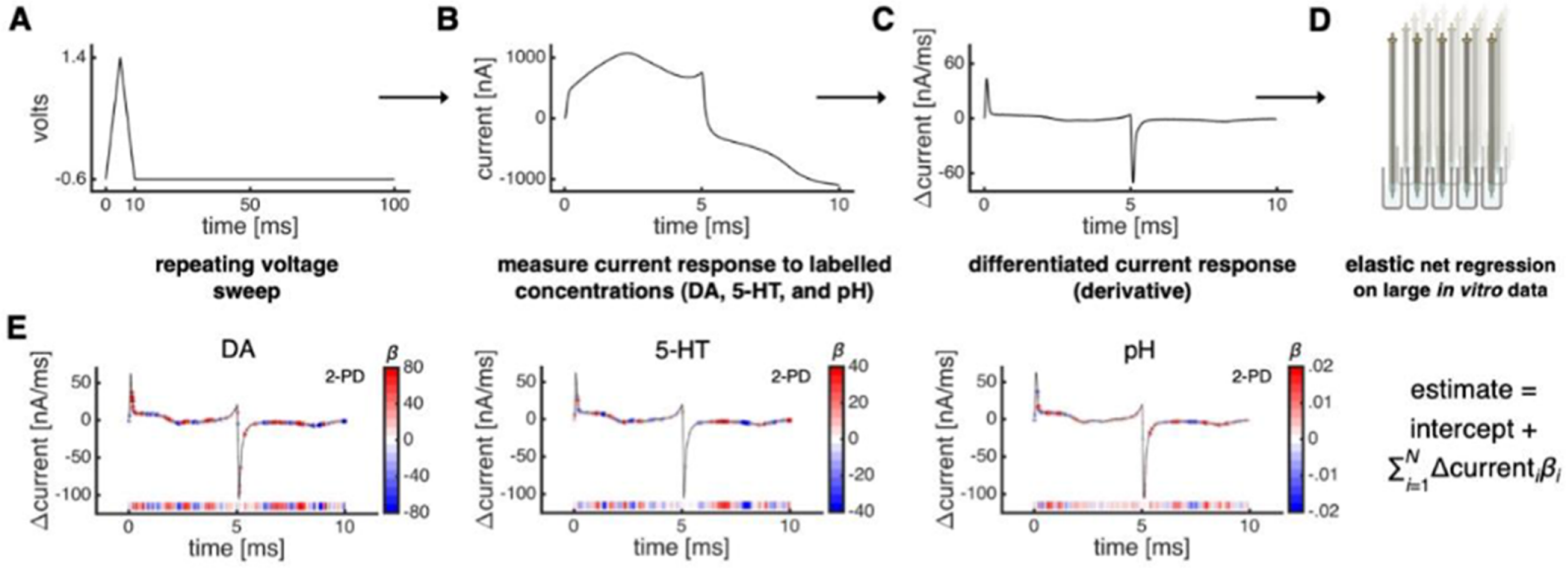
EN electrochemistry for dopamine (DA), serotonin (5-HT), and pH predictions. Reproduced with permission under a Creative Commons CC-BY license from Bang, D; Kishida, KT, Lohrenz, T; White, JP; Laxton, AW; Tatter, SB, Fleming, SM, Montague, PR. *Neuron* **2020**, *108*(5), 999–1010.

EN electrochemistry has been compared to FSCV-PCR.^85^ Theoretically, PCR and ridge regression are most closely related as both do not enforce sparsity. Experimentally, PCR is unreliable at low dopamine concentrations and confounds pH changes with dopamine predictions.^85^ Empirically, PCR and PLSR require less training time than EN due to the nature of the hyperparameter search space. Compared with PCR, supervised methods such as PLSR and regularization are less susceptible to reconstructing irrelevant features.

Furthermore, an argument can be made that model validation is not a signal reconstruction problem (as in the Q_α_ approach) but rather a test set predictive accuracy problem (i.e., the reconstruction accuracy of an input signal is unimportant as long as the model predictions perform well).^89^ Because regularization selects individual features rather than linear combinations, model training is less reliant on variations between points and more reliant on variations within points related to the target. While Q_α_ maintains that voltammograms containing unaccounted-for variance should not be analyzed at all, cross-validation and test set validation suggest that such variance has been trained to have a minimal effect on the model output. Regardless, both Q_α_ and test set validation can be confounded by interferents contributing similarly to the analyte signals.

### 2.3 Feature Engineering

Fast voltammetry data are preprocessed in several common ways before model training. We refer to these preprocessing procedures as feature engineering because they involve some combination of transforming, excluding, extracting, or otherwise manipulating data features prior to prediction.^93^ For example, the use of univariate linear regression can be considered manual feature engineering; the analyst decides which point (voltage) from the voltammograms to use for peak amplitude calibration.

Standardization, normalization, and derivative-based approaches are used to adjust the scale of voltammograms before model training.^57^ These processes improve the speed and accuracy of the training process and can be a required assumption of the underlying model or data distribution (e.g., mean-centered with unit variance). Most techniques utilize standardization (e.g., resulting in features with a mean of zero and standard deviation of one) or normalization (e.g., resulting in features ranging from negative to positive one). In contrast, others (such as EN electrochemistry) utilize the first derivatives (differentiated voltammogram traces).

Preprocessing can alter the weights of certain parts of voltammograms during model training by changing the relative magnitude of the responses. For example, normalization emphasizes the peak amplitude. Standardization emphasizes variability, and differentiation emphasizes the rate of change (i.e., peak and switching potentials).^26,57,85^ Output data (i.e., concentration) can be preprocessed for similar reasons, using *z*-scores for relative changes or a square root function to enforce non-negativity constraints.^82,88,89^

Various averaging and filtering techniques denoise, deconvolute, or otherwise filter data, usually via linear assumptions. These steps are often done after performing conventional background subtraction, which removes some (but not all) non-Faradaic current (discussed in greater detail below).^4^ For example, deconvolution and Fourier analyses can further remove non-Faradaic current^94,95^ and denoise data.^55,96,97^ Other linear transformations and various digital signal processing protocols continue to be developed for voltammetry-specific uses.^98,99^ The Hilbert transform has been applied to AC-voltammetry data to distinguish between serotonin and dopamine.^100^ An advantage of the Hilbert transform is that this technique does not rely on assumptions of data stationarity or linearity. Wavelet transformation has been used to preprocess phasic dopamine release traces and for compressive FSCV sensing, in which various domain transformations and reconstruction algorithms were compared.^101^ Efficient data compression is needed to develop wireless FSCV systems further due to sampling rate constraints.^102^

Pre-processing approaches can alter the weights of certain parts of voltammograms during model training by changing the relative magnitude of the responses.

One feature engineering choice commonly used in voltammetry techniques and models is background subtraction. The use of background subtraction vs background-inclusive data treatment is discussed in detail elsewhere.^4^ Background-inclusive voltammetry, paired with machine learning, enabled simultaneous recordings of phasic and tonic dopamine and serotonin, which few, if any other techniques have been able to do.^26,103,104^ The EN electrochemistry and rapid pulse voltammetry techniques are some of the few approaches that omit background subtraction. Indeed, with judicious algorithm selection, tedious preprocessing can be eliminated, and raw data can be utilized with minimal feature engineering. For example, deep learning methods, such as convolutional neural networks (CNNs; Section 4), excel at automatic feature engineering and perform better when trained with raw data rather than preprocessed data, as shown in fields such as vibrational spectroscopy.^105,106^

More computationally complex feature selection techniques have been explored for similar models in fields other than fast voltammetry (e.g., genetic algorithms).^107–110^ Feature selection techniques trade off potential information loss and overfitting for improved model performance at the added expense of computation time. Feature selection algorithms are typically implemented through a trial-and-error approach, as numerous techniques exist, but none are guaranteed to generalize effectively. A specific type of feature engineering that has been applied to fast voltammetry (best subset selection) is discussed in the context of transfer learning (Section 5.2). However, many feature engineering algorithms do not involve “learnable” parameters or do not directly relate to concentration quantification, so we do not cover them further here. Meanwhile, some models discussed above “learn” to perform feature selection (regularization) or extraction (dimensionality reduction) during the training process.^108,109^

### 2.4 Extensions of Linear Models

Generalized linear models (GLMs) are an extension of linear models that enable the underlying distributions of the outcome variables (e.g., analyte concentrations) to be assumed to be non-normally distributed. The pioneers of multiple-cyclic square wave voltammetry (MCSWV) utilized GLMs to predict tonic dopamine concentrations.^111^ This study demonstrated the power of probabilistic inference rather than peak-based signal analysis. Further extensions of GLMs should be explored, as these models could account for the temporal correlation between observations (adsorption/desorption between scans) and non-normal distributions, while addressing nonlinearities in the data.^112^

## 3 Nonlinear Methods

When might nonlinear methods be appropriate for voltammetry data? Based on electrochemical theory, the concentrations of analytes undergoing faradaic electron transfer are linearly proportional to the current generated.^39^ Thus, using a nonlinear relationship to model a known linear relationship is expected to be inefficient. However, there are cases in which nonlinearities arise in voltammetry. For example, multicomponent mixtures, such as dopamine, ascorbic acid, and divalent cations (Mg^2+^), exhibit nonlinear behavior.^78,113^ Temporal electrode drift is nonlinear, although including drift in training sets of linear models works well as a corrective measure.^66^ Regardless, the origin of such nonlinearities could include biofouling, varying experimental noise sources, surface phenomena affecting electrode responses over time, and other dynamic background changes.^4^ Only a handful of nonlinear models (excluding deep learning; Section 4) have been explored for analyzing fast voltammetry data.

### 3.1 Kernel and Tree-Based Approaches

Support vector machines make up a popular group of nonlinear models capable of classification and regression. Support vector machines transform the original feature space into a higher-dimensional space using a kernel, which is then used to create linear decision boundaries. These linear decision boundaries in transformed space are translated into nonlinear boundaries in the original space.^87^ Matsushita et al. used support vector machines to classify phasic dopamine release vs nonrelease. They achieved >95% accuracy on a public data set of 285 false-color plot FSCV traces from nine separate recording sessions in rats.^114^ Still, they required manual extraction and labeling to generate the training set.

One of the few reported applications of support vector machines in quantifying neurotransmitters from fast voltammetry data was for the detection of dopamine using FSCV.^115^ The authors showed that preprocessed PCA and raw (down-sampled) data could be classified and regressed using support vector machines. The PCA step was used to reduce the training time at the expense of a slight decrease in predictive accuracy. Interestingly, classification was first performed on the data to determine whether dopamine was present, and then regression analysis was performed.

Tree-based models segment the predictor variables using splitting rules, creating a tree-like structure. These methods are interpretable and straightforward. The simplest form is decision tree regression.^116^ Few studies have utilized tree-based approaches for neurotransmitter analysis.^117^ Fewer, if any, studies have used these models for voltammetry data. Bagging, random forests, and boosting approaches can be used to enhance predictive accuracy. These approaches are often referred to as ensemble techniques, as they combine multiple learners. Ensemble methods build a strong prediction model by combining weaker, simpler models, much like a random forest averages across a combination of many trees.^87^ When using random forests and boosting for phasic dopamine classification, an accuracy of ∼72% was achieved, which is below the performance of support vector machine and deep learning approaches.^118^

Head-to-head comparisons of fast voltammetry data analysis across various linear and nonlinear models are scarce. A report that compared linear, tree-based, k-nearest neighbors, and support vector regression (SVR) used differential pulse voltammetry to detect dopamine and serotonin in blood serum.^119^ Here, linear regression and support vector machines performed the poorest. One reason is that support vector machines move data into a higher-dimensional space, which is counterintuitive to the reason for applying dimensionality reduction. Perhaps for similar reasons, nonlinear dimensionality reduction techniques, including nonlinear extensions of PCR and PLSR, are also underreported. Another reason for the dearth of nonlinear approaches is poor interpretability, aside from tree-based approaches, which are among the most interpretable models (Section 6).

A separate report compared multiple preprocessing methods and models (linear and nonlinear) for detecting ascorbic acid, uric acid, dopamine, and nitrate. The study used a glassy carbon electrode and square wave voltammetry for an application in human serum.^120^ Interestingly, the authors found that a nonlinear modification of PLSR, utilizing a radial basis function, performed best overall. By contrast, artificial neural networks (Section 4) were less accurate and required ∼100 times greater computational time. Fast voltammetry will benefit from large-scale, head-to-head comparisons of various models using neurochemical-specific data sets for brain applications. No single model or class of models has yet been demonstrated to work well across all voltammetry techniques, even for similar classes of analytes.

One of the most comprehensive comparisons of machine learning models for fast voltammetry was conducted by Goyal and coauthors, who utilized MCSWV to detect tonic (unstimulated) concentrations of dopamine, norepinephrine, and serotonin.^37^ These authors compared the performance of PCR, PLSR, SVR, and regularization (ridge, LASSO, and EN) with a deep learning approach (discussed below). Using a within-electrode analysis, the SVR model dramatically outperformed LASSO, EN, and PLSR. The PCR and ridge methods performed the worst, and the deep learning approach performed the best overall. Here, PCA was performed before regularization to reduce computational time, and PCR/PLSR components were chosen by using the variance threshold technique. Both of these approaches could bias the performance toward worse-than-expected results unless they are tuned in a more careful, yet time-consuming manner. Notably, SVR likely performed better for MCSWV data because this type of voltammetry is inherently a higher-dimensional technique due to the waveform design. While the deep learning approach outperformed SVR across electrodes, the other shallow learning algorithms that performed worse within electrodes were not tested across electrodes.

## 4 Deep Learning

Deep learning has garnered interest due to its ability to model complex nonlinear relationships. Deep learning refers to a subset of machine learning models that utilize artificial neural networks with many layers to learn representations from large data sets. Due to its recent attention and impact, we cover deep learning in its own section. Excellent introductions to deep learning and artificial neural networks can be found elsewhere.^121,122^

The ability to perform automated feature extraction is a key advantage of deep learning. A disadvantage is the potential for overfitting and high computational cost when a network is fully connected (i.e., all neurons in one layer are connected to all neurons in the next layer). Among other techniques to address these problems, CNNs have emerged as a leading architecture (i.e., connectivity pattern) for high-dimensional data, such as image data.^123^ Given that voltammetry data are high-dimensional data with short- and long-range correlations (i.e., a single analyte can have redox peaks across many voltages), CNNs are a good candidate architecture to analyze voltammetry data. Other types of layers are used in conjunction with convolutional layers, such as pooling (which can help with the translational invariance associated with small shifts in redox potentials) and dropout (to reduce overfitting).

Early applications of artificial neural networks to FSCV data focused on classifying phasic dopamine release using CNNs.^118,124^ As noted earlier, the manual analysis of false-color plot data for phasic neurotransmitter release is time-consuming and lends itself to automation. Matsushita et al. reported accuracies exceeding 98% for classifying over 2000 FSCV traces of 20 s recordings with and without electrically evoked ventral tegmental area dopamine release across 35 mice in two different laboratories.^118^ The authors explored architectures, such as a ten-layer CNN, an Inception v3 network, and YOLOv3. Patarnello et al. later achieved 98% accuracy using an ensemble of 60 AlexNet ensembles, each with ∼8 layers.^124^ Al-Haija et al. reported 99% accuracy across 6030 traces using data augmentation and DarkNet, a CNN architecture with ∼24 layers.^125^

The first applications of deep learning to predict neurotransmitter concentrations from fast voltammetry data have emerged in the last several years. Again, one of the earliest reports was for dopamine detection.^126^ Here, the authors focused on implementing deep learning using less resource-intensive hardware (a field programmable gate array) for integration into wearable and wireless hardware (a hardware-software codesign). To reduce computational resource requirements, a CNN was not used. Instead, a fully connected network was trained and then “pruned” to reduce the number of parameters.

The deep learning model was trained to recognize concentration and the electrode used (as defined by the length of the cut tip of the carbon fiber) from the voltammograms as input (a “multitask” approach). The goal was to classify voltammograms with discrete concentrations of 0, 0.5, 1, 1.5, and 2 μM dopamine and to identify from which electrodes voltammograms were acquired; continuous regression was not carried out but noted as future work. The authors then reduced the number of parameters by setting thresholds in a custom pruning algorithm approach. They reduced the parameters by a factor of 3 (from 166,102 to 52,193), while achieving slightly higher accuracy (from 96 to 97.2%) after retraining. The performance of the pruned artificial neural network in concentration identification was compared with PLSR and PCR. The deep learning approach was more accurate for test set accuracy across all five electrodes, and PLSR outperformed PCR.^126^

Xue and co-workers were among the first to utilize an autoencoder architecture for FSCV (Figure 8).^127^ Autoencoders consist of encoder and decoder functions, typically modeled by artificial neural networks (here, CNNs). The output of the encoder, and thus the input of the decoder, is a latent feature representation of the original data.^128^ The autoencoder was trained to predict the dynamics of ascorbic acid, dopamine, and NaCl concentrations. The authors noted that nonfaradaic sensing could be accomplished, as shown for NaCl, because autoencoders account for nonlinearities that methods such as PCR cannot.

**Figure 8:**
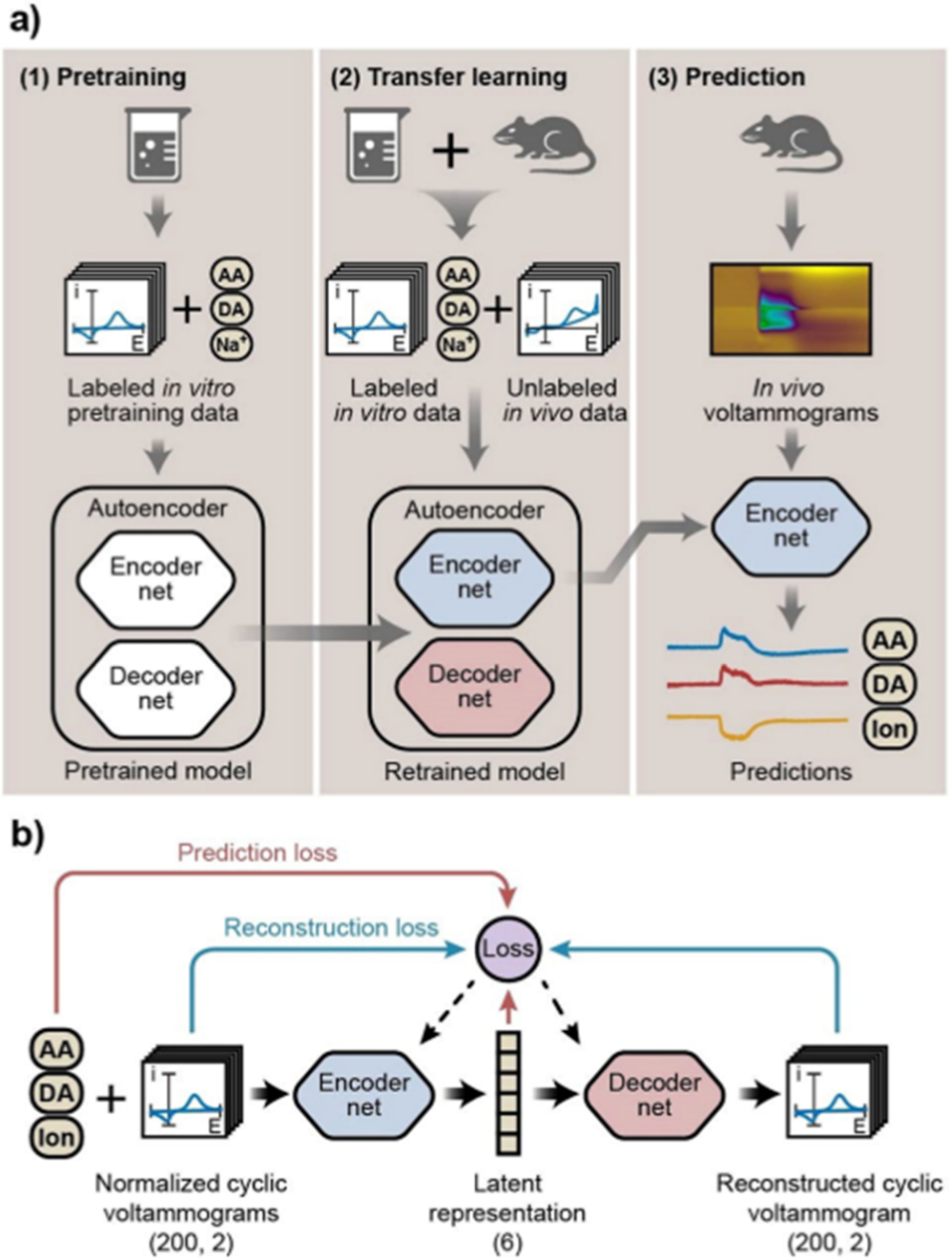
Use of an autoencoder with semisupervised learning for fast scan cyclic voltammetry (FSCV). AA = ascorbic acid, DA = dopamine, and Na^+^/ion = sodium chloride. Reproduced with permission from Xue, Y; Ji, W; Jiang, Y; Yu, P; Mao, L. *Angew Chem Int Ed* **2021**, *60*, 23,777. Copyright © 2021 Wiley-VCH GmbH.

Notably, this approach used an autoencoder with transfer learning (Section 5.3). In this process, the authors trained the full model (consisting of an encoder and decoder) using labeled in vitro data, as was done in most cases above. The loss function was defined to minimize the error between predicted and actual concentrations, with an additional term for reconstruction loss. In other words, the encoder takes high-dimensional voltammograms as input and outputs the encoded, low-dimensional concentrations, while the decoder uses these concentrations to reconstruct the original voltammograms. This model is referred to as “pretrained”, as it has not been presented with its specific task or the domain on which to perform inference (in vivo).

The pretrained model (encoder and decoder) is then retrained using labeled in vitro training voltammograms and unlabeled in vivo voltammograms. In this process, the model is trained to minimize reconstruction loss on both labeled and unlabeled data while accurately maintaining the concentrations of the labeled data. As a result, the decoder enables the encoder to preserve relevant features common to both in vitro and in vivo data. This approach is commonly known as semisupervised or transfer learning, as the data are partially labeled and partially unlabeled (see Section 5).

The concentration predictions are then made by using only the fully trained encoder portion of the model (Figure 8). By minimizing both types of loss, generalizable feature extraction is improved, while overfitting is reduced. Here, the authors partly attribute model success to the stability of the vapor-grown carbon fiber electrode. The final model predicted μM fluxes of ascorbic acid and dopamine, as well as mM NaCl fluxes, in vitro and in vivo. For validation, these authors microinfused KCl to induce spreading depression in the rat striatum and 4,4-diisothiocyanostilbene-2,2′-disulfonic acid to measure the inhibition of Cl^–^ influx. Lastly, the autoencoder approach enabled the reconstruction of voltammograms to be studied through the decoder network, providing a means for statistical validation similar to the use of Q_α_ for PCR. The Mao group later reported the use of a variational autoencoder to separate the faradaic (ascorbic acid and dopamine) and nonfaradaic (NaCl) contributions to voltammograms for improved in vivo performance during ion fluxes.^129^

Mena and co-workers combined deep learning with fast-scan controlled adsorption voltammetry (FSCAV) for serotonin detection to quantify in vivo concentrations.^130^ The FSCAV technique is derived from FSCV, but it utilizes a combination of high-frequency waveform scans and extended holding potentials. Challenges in postexperiment calibration, the nonlinear effects that occur postimplantation, and variable sensitivity across electrodes when using FSCAV inspired the use of deep learning for this application.

Two shallow artificial neural networks were constructed, each comprising two hidden layers. The first network predicted serotonin concentrations solely on the basis of four voltammogram-specific characteristics, utilizing training data collected from a single electrode or 140 electrodes. While both neural network methods outperformed linear regression in predicting serotonin, the training approaches for the two neural networks performed comparably. This result was surprising because deep learning models are generally believed to improve with more training data.

Another network was constructed using all training voltammogram data (1100 sampling points per voltammogram instead of four input characteristics) from all 140 electrodes. This model performed comparably to the previous neural networks, provided consistent experimental procedures were followed, and it circumvented the need for electrode postcalibration. The authors attributed their success to the correlation between the background current and electrode sensitivity, as inferred from the inclusion of the full set of data from information-rich voltammograms. The model was validated in vivo in the mouse hippocampus using escitalopram and lipopolysaccharide to alter serotonin levels. The calibration-free results were similar to those obtained with more cumbersome manual methods.

Rather than predicting phasic or tonic concentrations, a different data analysis approach, as used by Buchanan et al., predicted the ratio of serotonin to dopamine using a CNN.^131^ Here, the authors investigated the cotransmission of serotonin and dopamine in the mouse hippocampus. The authors used labeled in vivo color plots of pharmacologically verified, electrically evoked release of striatal dopamine or hippocampal serotonin to train the model. Data augmentation was used to create additional labeled synthetic training data by summing known, randomized ratios of the verified color plots. The authors then generated training sets of 5000 color plots per animal to predict serotonin-to-dopamine ratios. Their findings suggest that l-DOPA treatment redirects the synthesis of serotonin to dopamine in serotonergic neurons projecting to the hippocampus.

Choi and colleagues reported one of the first direct comparisons of FSCV data analysis with PCR vs deep learning (Figure 9).^36^ For the PCR model, the components were chosen to explain 99.99% of the variance, and Q_α_ was used to determine valid (as defined by the reconstruction error) voltammograms. A VGGNet-inspired CNN architecture was used for deep learning with 5-fold cross-validation. Dopamine, norepinephrine, epinephrine, serotonin, and pH predictions were compared in single and multianalyte combinations across five electrodes by training in a flow cell. No significant differences in accuracy were found between PCR and deep learning for single-analyte detection. For multianalyte mixtures, however, deep learning was more accurate (by up to 20%) compared to PCR. This in vitro result supports the use of CNNs vs. a linear PCR model in discerning complex mixtures of neurotransmitters, presumably due to the ability of CNNs to model nonlinearities that may arise from multianalyte or multielectrode effects.

**Figure 9:**
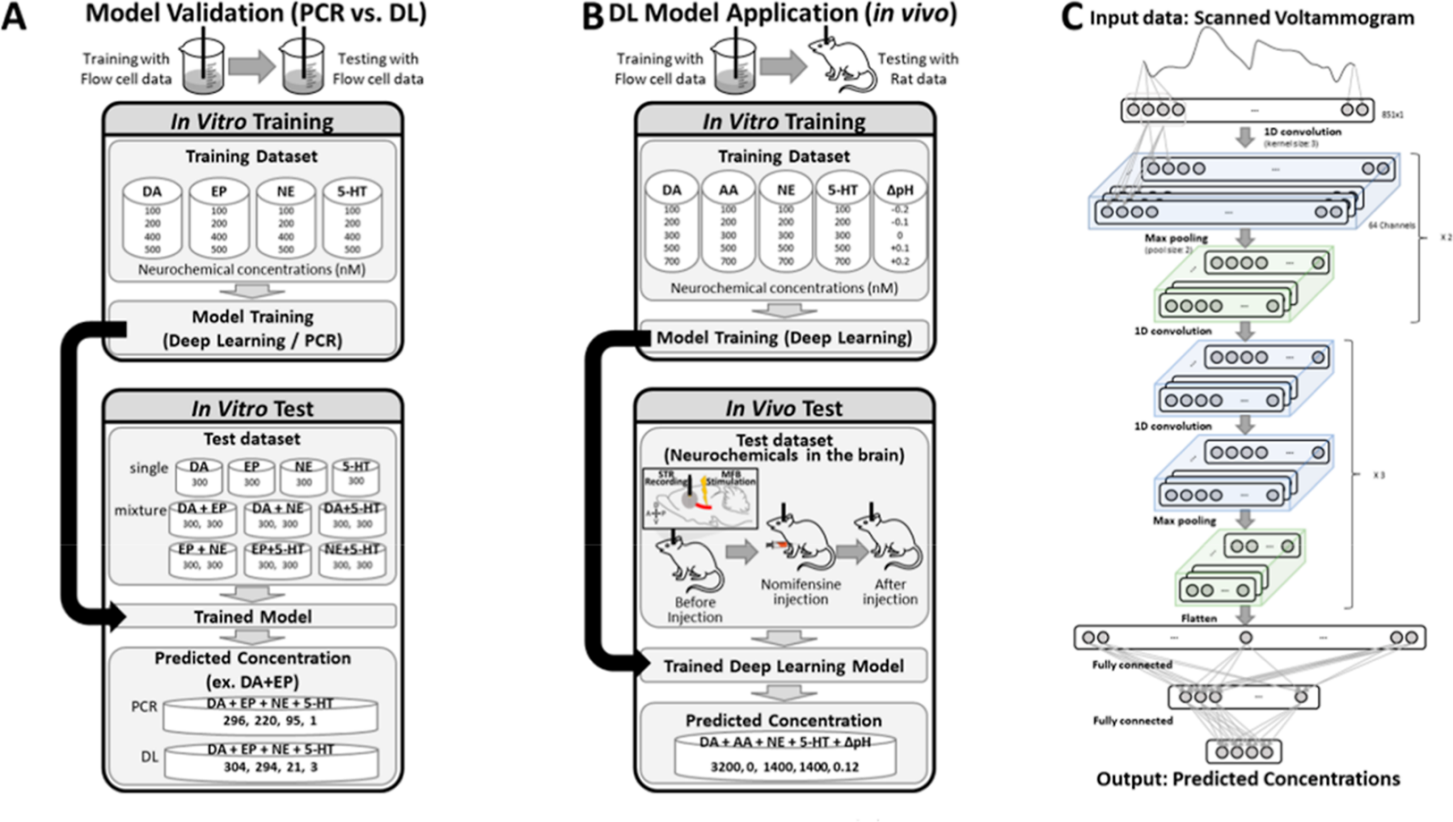
Comparison of PCR and deep learning (DL) for FSCV. DA, EP, NE, 5-HT = dopamine, epinephrine, norepinephrine, and serotonin, respectively. Reprinted (adapted) with permission from Choi, H; Shin, H; Cho, HU; Blaha, CD; Heien, ML; Oh, Y; Lee, KH; Jang, DP. *ACS Chemical Neuroscience* **2022** *13* (15), 2288–2297. Copyright 2022 American Chemical Society.

In vivo validation was performed by stimulating the medial forebrain bundle and monitoring phasic dopamine release in the dorsomedial striatum of anesthetized rats, with nomifensine as a pharmacological challenge.^36^ As expected, the level of stimulated dopamine predicted by deep learning increased significantly after nomifensine administration. The pH predictions during electrical stimulation were also significantly increased by approximately +0.1 units compared to predrug administration. Serotonin, ascorbic acid, and norepinephrine concentrations were not predicted to change significantly. The dopamine results tracked with conventional linear postcalibration. The PCR models trained on more complex mixtures outperformed single-analyte training sets used in earlier applications, supporting the increasingly common use of large, diverse training sets.

The authors noted the limitations of incomplete training set designs on both PCR and deep learning predictions.^36^ Analytes and interferents not present in the training set but present in the brain limit the in vivo generalization of any model. While PCR uses Q_α_ to identify voltammograms with possible contributions from nontrained analytes, no such approach was employed for deep learning in this report. The approach by Xue et al. above enables signal reconstruction from a deep learning model and addresses this reliability concern.^127^ In summary, deep learning performed as well as, if not better than, conventional linear methods for fast voltammetry in vitro and in vivo.

Twomey et al. recently reported a comprehensive comparison of different artificial neural network architectures.^86^ They compared “out-of-electrode” (i.e., making predictions from voltammograms obtained from a different electrode than the one used for training voltammograms) performance across a data set comprising 76 electrodes for detecting dopamine, serotonin, and norepinephrine. The architectures included two fully convolutional networks, two MLP models of varying size, an InceptionTime network (an ensemble of CNNs based on a specific architecture), and two domain-specific architectures (SSVEPformer and EEG-Transformer) adapted for voltammetry. The InceptionTime model was the best-performing model, but it was also one of the most susceptible to artificially induced electronic noise. The MLP was the next best performer and was the least susceptible to noise. Noise was induced by measurements taken outside a Faraday cage with commercial electronic laboratory equipment nearby. These findings suggest that nonconvolutional architectures may be useful for FSCV data. (Fully convolutional networks performed worst in terms of noise and accuracy.)

Additionally, in Twomey et al., larger models (with more parameters) did not necessarily improve performance within the architectures studied.^86^ Model performance was correlated across analytes, with serotonin predictions being the best indicators of overall performance. The MLP was susceptible to noise because it uses derivatives for preprocessing. Reducing noise at the source was deemed important in preprocessing, as noise can have significant adverse effects on the model. Thus, some manual feature engineering or ensemble averaging may be needed for deep learning. However, eliminating all types of noise is nearly impossible, especially in an in vivo environment.

Lastly, these authors demonstrated a method for identifying “deviant” probes (i.e., electrodes that fail to generalize or produce inaccurate predictions) by investigating model embeddings. Such an approach, among others, will help aggregate large, multielectrode training sets for out-of-electrode predictions. Following the in vitro success of the InceptionTime architecture described above, the Montague group utilized a modified version of the InceptionTime CNN model for detecting noradrenaline in the human amygdala,^35^ which is an ensemble of multiple models. These authors predicted concentrations of serotonin, dopamine, and norepinephrine.

Another approach extended a previously developed technique, second-derivative-based background drift removal, to predict tonic dopamine and serotonin using deep learning.^132^ The authors used a temporal convolutional network. While results were only demonstrated in vitro, simultaneous analyte detection across time scales remains highly sought after.

Initial reports of tonic measurements (∼10 s temporal resolution) using deep learning^37^ were inspired by the autoencoder approach developed by Xue et al.^127^ Aside from predicting tonic concentrations through the use of MCSWV and employing 2D instead of 1D convolutional blocks, this autoencoder (named DiscrimNet) was trained on data sets across electrodes, rather than within a single electrode. Across-electrode training paradigms are hypothesized to generalize better in vivo, where electrode conditions are significantly altered but the model retains conserved salient features. Furthermore, a key benefit of this across-electrode model is that it does not require retraining for additional electrodes and can instead be reused in real time as voltammetry data are being acquired.

The possibility of avoiding retraining has direct applications to closed-loop deep brain stimulation (Section 4.2). When in vivo data obtained in anesthetized rat nucleus accumbens using naïve electrodes (not included in the training set) were analyzed by DiscrimNet, tonic concentrations of dopamine, serotonin, and norepinephrine followed historical trends and pharmacological challenges to cocaine and oxycodone.^37^ The authors attributed success to the higher-dimensional data obtained using MCSWV for tonic rather than phasic measurements (especially to aid in differentiating dopamine and norepinephrine), as well as the semisupervised approach performed across, rather than within, electrodes. Future work is needed to optimize DiscrimNet for handling awake, behaving subjects and chronic implantation artifacts.

Across-electrode and semi-supervised training paradigms are hypothesized to better generalize out-of-electrode and in vivo, in which electrode conditions are significantly altered but the model retains conserved salient features.

### 4.1 Are Big Data Required?

A commonly accepted prerequisite for deep learning is that large data sets are required. Interestingly, while some groups have achieved success with deep learning across large, multielectrode data sets, the use of artificial neural networks for calibration-free FSCAV did not yield an improvement with a large data set.^130^ Response deviations across electrodes when combining data sets might have contributed. Advanced methods are being developed to improve the subsets of across-electrode data sets that should be combined to create multielectrode models (Section 5.1). Other fields are also questioning the “bigger is better” mentality when it comes to machine learning data set size.^133^

Generally, voltammetry users are increasingly adopting the latest machine learning advancements and developing techniques that capture or integrate as much chemical information as possible. Recent deep learning networks can have layers ranging from a couple to more than a dozen, with the number of parameters surpassing half a million. However, the number of electrodes, voltammograms, and calibration concentrations and interferences used to train these models varies significantly. Some reports utilize dozens of electrodes across tens of thousands of voltammograms and a wide range of concentration calibration mixtures. Others have found success using much simpler approaches, involving only single electrodes, a few calibration samples, and a limited number of training voltammograms. This ambiguity regarding “how much training data is required?” partly stems from the absence of standardized data sets, evaluation metrics, and challenges in assessing ground truth in vivo.

### 4.2 Data Fusion, Devices, and Auxiliary Uses

An exciting area of progress in biosensing, including voltammetry, is the integration of data fusion and multimodal sensors.^20^ Data fusion can be homogeneous (i.e., combining data from the same sensing mechanism) or multimodal (combining data from different sensing mechanisms).

Fused homogeneous data can come from a single sensing modality, such as voltammetric electrode arrays,^23,24^ in which each electrode can have varying materials, coatings, or waveforms. This concept extends soft sensing and creates a voltammetric “electronic tongue” for the brain.^134^ One can envision a scenario where multiple waveforms are applied, each with its own strengths and weaknesses. Ensembled corrections and improvements are made when data or predictions are fused (especially across materials). Some models may be better suited for specific analytes or sensing tonic or phasic release or could be more tolerant to fouling, drift, or noise. An ensemble of models that can vote on the best concentrations could be useful for future work.

Multimodal data fusion combines different sensing modalities. For example, field-effect transistor measurements were recently combined with voltammetry at carbon–fiber microelectrodes to determine pH.^135^ Other instrumentation and techniques combined with voltammetry include electrochemical impedance spectroscopy,^136^ iontophoresis and electrophysiology,^137,138^ functional magnetic resonance imaging,^139^ and optogenetics.^140^ As these techniques and devices continue to develop, training combined data sets or models could leverage the mutual information content.

Voltammetry is conducive to hardware miniaturization and portability, as well as on-device computing.^141–143^ This has led to voltammetry being a key component in developing closed-loop deep-brain stimulation systems. Such a system would require a device that can measure neurochemicals via voltammetry, analyze the data, make decisions using on-device computing, and respond with optimized electrical stimulation settings to create an Observation-Orientation-Decision-Action loop (Figure 10). Machine learning will play a key role in these devices to quantify neurochemicals using the techniques discussed in this review and for refining stimulation parameters. Several systems are being developed for this purpose.^144–148^

**Figure 10:**
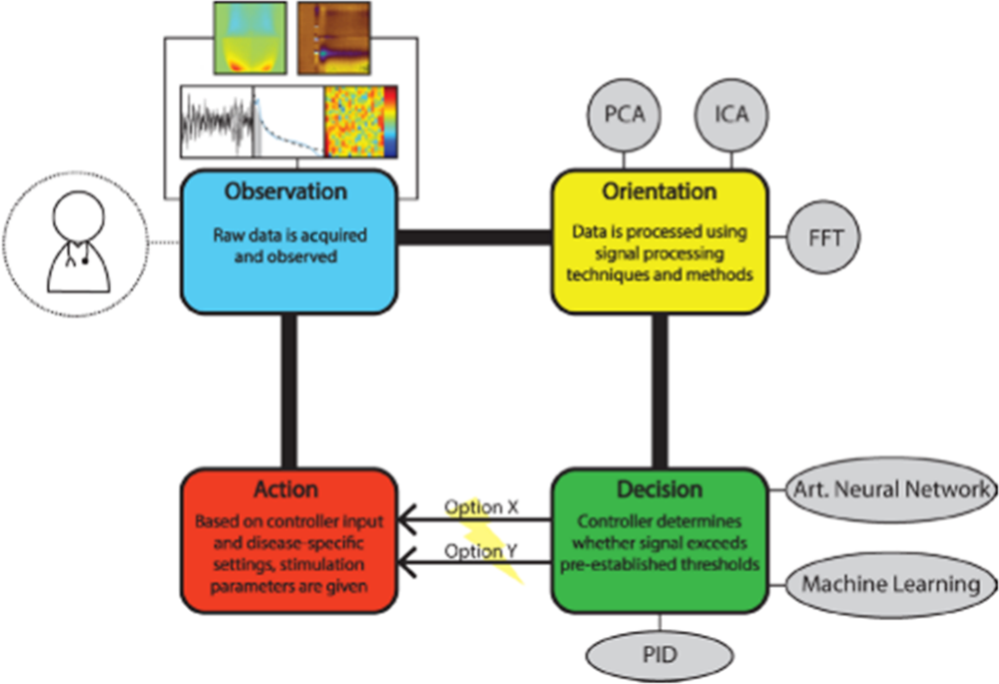
Observation-Orientation-Decision-Action loop. Reproduced with permission under a Creative Commons Attribution 4.0 International License from Rojas Cabrera, JM; Blair Price, J; Rusheen, AE; Goyal, A; Jondal, D; Barath, AS; Shin, H; Chang, SY; Bennet, KE; Blaha, CD; Lee, KH. *Reviews in Analytical Chemistry* **2020** *39* (1), 188–199. Copyright © 2020 Juan M. Rojas Cabrera et al., published by De Gruyter.

The use of machine learning to develop new voltammetry sensors is unexplored. Other fields have used machine learning to discover new materials and sensors.^149,150^ Fast voltammetry may also benefit from the machine-learning-inspired design of electrode materials. The panels of analytes monitored by fast voltammetry are continually expanding along with the development of new materials, waveforms, and models. Using data analysis with feedback control to alter waveforms may also see continued growth.^151^ For example, we reported a Bayesian optimization workflow for machine-learning guided design of novel voltammetry waveforms.^152^ As done in other fields, connecting these advances in feedback discovery loops could accelerate the development of new voltammetry approaches.^149,153^

Beyond the use of machine learning directly for voltammetry data analysis, there are uses of machine learning for auxiliary or complementary experiments. For example, hidden Markov models have been used to estimate correlations between pupillometry and noradrenaline.^35^ Computer vision approaches have been used to relate facial expressions of head-fixed mice to brain activity and emotional encodings,^154^ and reinforcement learning has been used to study reward processing.^155^

## 5 Transfer Learning

Several issues arise when applying in vitro calibration to predict outcomes from in vivo test data. Because the training and test sets are collected in different environments (i.e., domains) or come from different underlying statistical distributions, generalizing a model trained in vitro to perform inference on in vivo data presents challenges for brain voltammetry. The ground truth of analyte identity and concentration by voltammetry in vivo is currently unknowable. Obtaining in vivo training data is difficult, if not impossible, in some cases (discussed below). Moreover, it is often a statistical requirement that the test data are obtained in the same environment as the training data. While attempts to mimic some in vivo conditions, such as using postfouled electrodes and/or various buffer and interferent conditions, can help, many electrode surface-chemistry changes that occur in vivo cannot be replicated in vitro. This is termed a generalizability issue, a beaker-to-brain issue, or an out-of-distribution effect. These issues are the chief concerns when applying machine learning to in vivo voltammetry.

These issues are referred to as a generalizability issue, a beaker-to-brain issue, or an out-of-distribution effect and are the chief concerns when applying machine learning to in vivo voltammetry.

There are many possible origins for out-of-distribution shifts. For instance, even though a flow cell simulates release and reuptake kinetics, the brain is not “flowing.” Flow cells do not exhibit the same tortuosity and impedance environments as brain tissue. Additionally, the brain is subjected to intracranial pressure and is maintained at body temperature. It contains dissolved gases, along with many other differences that are either cumbersome or impossible to replicate in vitro or even ex vivo. While efforts are underway to mitigate electrode shifts at the source, such as developing innovative electrode coatings and configurations,^156,157^ no technique can yet truly mimic the brain, limiting many methods to validation in more controlled, replicable, or indirect environments, such as blood, urine, or sweat.

The issue of combining, training, and performing inference across multiple data sets or domains is not unique to voltammetry. Broadly, this field is termed transfer learning. Transferring model calibration across domains, such as different instruments and laboratories, has its roots in spectroscopy and chemometrics.^158^ There is a rich literature on transfer learning, domain adaptation, and domain generalization, as well as specific guidelines for chemometric applications.^159^ Many applications in the biological and medical fields face similar challenges where the distributions of training and test data differ. Voltammetry can benefit from the adoption of complementary approaches. Approaches developed for voltammetry and other techniques are detailed below.

### 5.1 In Vitro Multielectrode Models

Here, the training data are the source domain (beaker and flow cell) used to make inferences about the target domain (brain) data. Although the task remains the same between the source and target domains (neurotransmitter quantification), the domains differ. The failure of training sets to generalize in vivo occurs because our statistical learning algorithms assume that training and test data are from the same domain. This does not mean that using machine learning for in vivo voltammetry is unjustified. However, predictions are estimates and must be interpreted carefully, even for simple univariate analysis.^5,160^ The less the source and target domains differ, the more likely the models are to generalize.

One solution to model building that generalizes across domains is to obtain larger aggregated training sets across multiple electrodes and analytes, thereby producing a generalized representation over time. Large, multielectrode data sets capture variations across electrodes, resulting in models that generalize to unseen electrodes. As an extension, we can assume that a model can also generalize to in vivo domains because in vivo environments impact electrode performance such that each electrode can be considered a “different” electrode when used in the brain vs. the beaker (or flow cell). In vitro training sets are becoming larger and include more variations through multiple electrodes, analytes, and concentration levels to account for shifts in the marginal or covariate current distributions across a waveform. Along with postfouled calibrations, we can bridge source and target domains (Figure 11).

**Figure 11:**
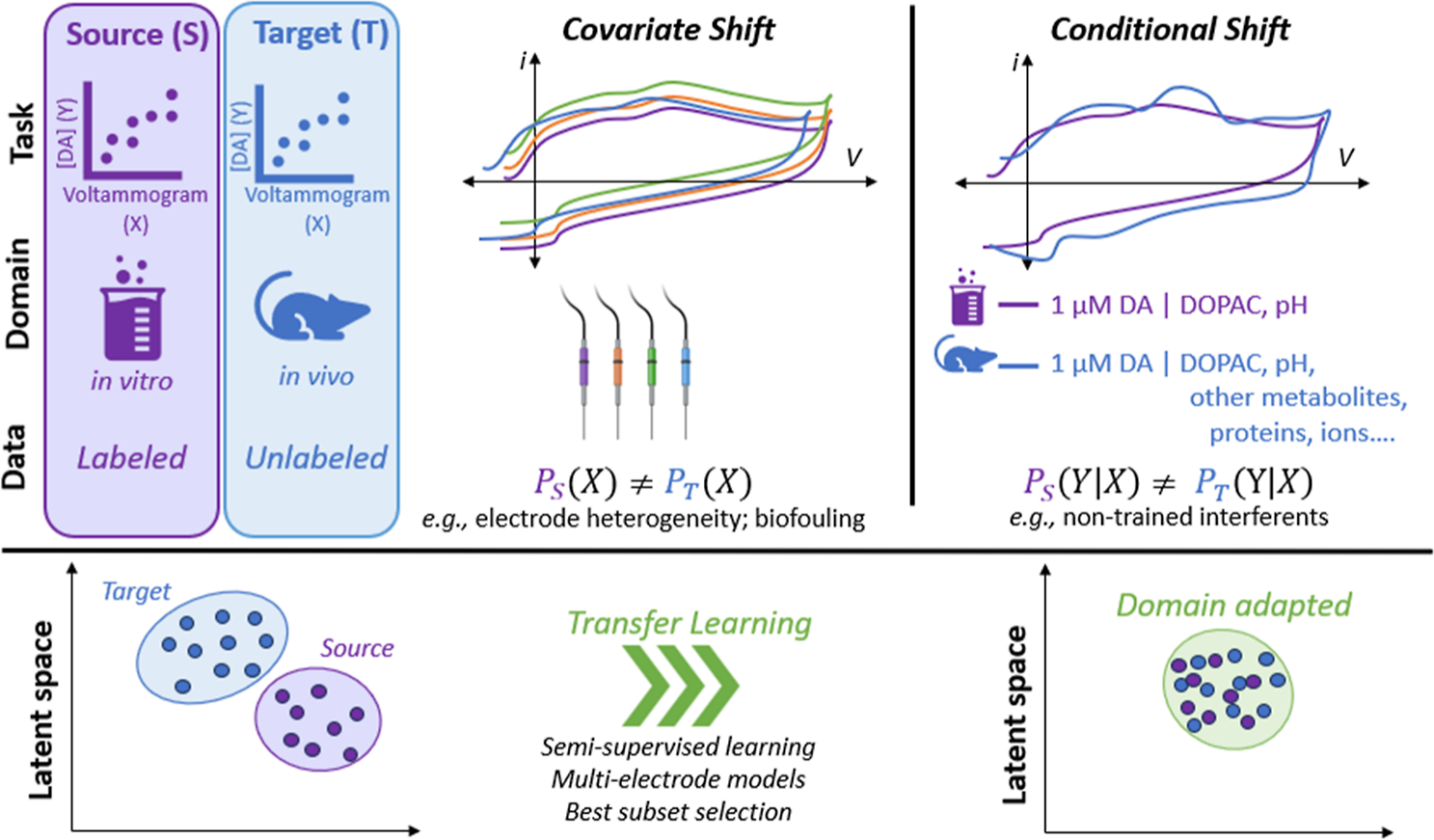
Transfer learning concepts in fast voltammetry.

Nonetheless, voltammetry microelectrodes are highly heterogeneous. Even within laboratories, they are almost exclusively handmade with considerable variations in performance characteristics, even when made by the same individual. Moreover, different research groups use different fabrication and testing protocols. There are no standardized voltammetry electrode metrics that have been agreed upon by the research community. Models trained on data acquired from one electrode will often fail to accurately predict test data from a different electrode, whether used in vitro or in vivo. Significant calibration differences also occur between prefouled training and postfouled predictions.^161^

While new electrode materials, fabrication techniques, and standardized testing metrics will help mitigate the impact of the generalization issue,^12,25,70,162,163^ the underlying statistical problem is that training voltammograms for each electrode and the corresponding neurotransmitter concentrations are derived from different marginal and conditional distributions, respectively. This is the root cause of poor generalizability. Optimal training sets may lie in a subset of the total available aggregate of training data. How should multielectrode data sets be combined to ensure generalizability (i.e., make predictions on electrodes not included in training, in separate domains, or both)?

One approach is to use subsets of multielectrode data sets. When training across multiple electrodes (and especially when aiming to generalize to electrodes outside the training set (i.e., out-of-electrode samples)), voltammograms generated across electrodes can exhibit response variations due to inherent differences in fabrication. Data subsets can be identified where only similar electrode responses to input variables are used to train the underlying model. This idea is supported by previous reports in which subsets of electrodes and measurements were highly correlated for within- and between-subjects/electrode training.^61^

Further, if the biology under investigation can be interpreted through relative changes (e.g., z-scores), the over- or underestimate of transient concentration changes becomes less of an issue. Kishida and coauthors began matching training data across electrodes by comparing shape similarities, which they found improved prediction accuracy and generalizability.^85^ To find similar voltammograms, unsupervised learning approaches, such as hierarchical clustering, can be used to identify data from electrodes with similar responses. Training sets across these electrodes are then combined into a single training set. A later approach by Loewinger et al. used covariate profile similarity (CPS) weighting (Figure 12).^82^ The authors leveraged the fact that covariates (i.e., voltammograms) from the target domain are available before modeling, such that unlabeled information can be leveraged similar to the semisupervised transfer learning approach used by Xue et al.^127^

**Figure 12:**
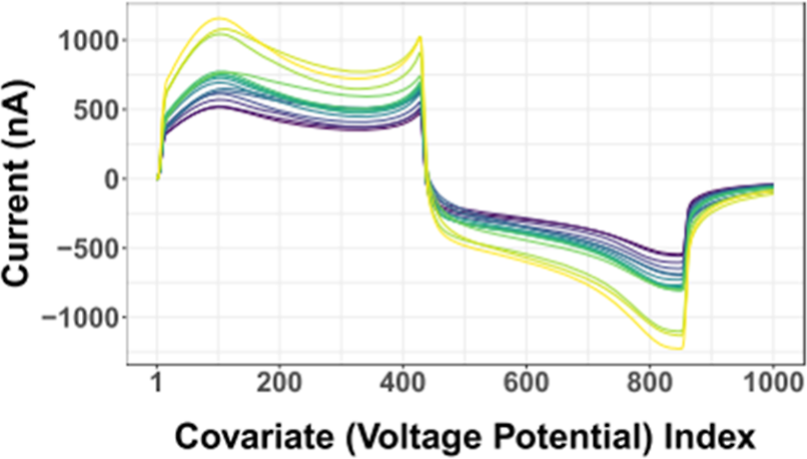
CPS weighting. Adapted with permission under a CC-BY-NC-ND 4.0 International license (https://creativecommons.org/licenses/by-nc-nd/4.0/) from Loewinger, G; Patil, P; Kishida, KT; Parmigiani, G. *bioRxiv* **2021** 856385; doi: 10.1101/856385.

A different approach involves creating multiple models for specific concentration ranges.^85^ This is done by selecting training data that are normally distributed around a concentration range of the interest. Models trained on narrow concentration ranges performed better in multiple use cases. This approach allows for “guess and check”, where specialized models for various concentration ranges can be utilized to analyze unknown in vivo data, such that appropriate models and concentration ranges can essentially be searched for. The downside is the substantial computational effort needed to train and apply such models.

An interesting development of novel statistical learning methods specifically designed to address fast voltammetry data set ensembles, detailed by Loewinger and co-workers, is called the “study strap ensemble”.^82^ Here, “studies” refers to in vitro training sets of dopamine using FSCV, each acquired at a different electrode. Multistudy learning thus refers to the training of statistical inference models on data sets that combine data from multiple electrodes. Historically, this has been accomplished either by pooling all studies together and fitting a single model to the merged data set (training on the merged data set; TOM). Alternatively, separate models are trained on individual data sets, and the predictions are averaged (observed-studies ensemble; OSE). The study strap combines both approaches to find the ideal combination of studies using custom-developed algorithms and a tuning parameter that allows fine control over heterogeneous combinations of data sets. The study strap can also be combined with CPS weighting, allowing only similar studies to be combined.

The study strap approach has been evaluated using electrodes not included in the training data as a proxy for electrodes in the brain, where data distributions shift significantly, making the data appear to have been collected from a “different” electrode. Performing well across unseen electrodes is a good indicator of in vivo generalizability. Here, PCR was used rather than regularization, as the former offered superior cross-study performance. Per an EN electrochemistry protocol, preprocessing included the voltammogram derivative but not background subtraction. The TOM, OSE, and study strap approaches were compared across 15 studies (comprising a total of 20,000 samples) with and without CPS. The improvements of CPS and study strap methods developed in this work matched or outperformed the standard approaches of combining multiple data sets, such as those by TOM and OSE, with improvements in root mean squared error of dopamine predictions ranging from 5% to 60%.^82^ Study strap algorithms were released as an open-source package. However, these methods are computationally intensive.

### 5.2 Best Subset Selection

Instead of finding the optimal combination of data sets to create generalizable fast voltammetry models, a separate approach is to find the optimal combination of model features that generalize to unseen data, such as in best subset selection. Best subset selection refers to identifying a subset of features from the total set of features to create a model that reduces prediction errors and enhances interpretability.^87^ While best subset selection is related to feature engineering and model selection, its applications in fast voltammetry are limited and primarily associated with domain adaptation applications.

Best subset selection refers to finding a subset of features from the total set of features to create the model to reduce prediction errors and enhance interpretability.

In separate work, Loewinger et al. extended the best subset selection approach to multitask learning. Here, analyte predictions at separate electrodes were considered separate tasks,^164^ as the marginal distribution of electrode signals, and hence the conditional distribution of the concentrations, differed across electrodes. For multielectrode training sets to be useful, a model should be able to conserve the useful features of voltammograms even when the data are heterogeneous and noisy.

One common solution is to enforce sparsity through a penalized error term, as done in regularization.^164^ Here, solutions were identified that leveraged data patterns and individual data values. For example, each electrode may have a different pattern of features (i.e., area of analyte activity at the electrode surface) specific to that electrode. Generalizing across electrodes finds conserved patterns across the electrodes. The authors called this approach “support heterogeneity regularization”. Compared with conventional methods for training across multiple electrodes, this new technique outperformed all others in predictions on a held-out electrode. The code to perform multitask learning was also released as an open-source package.^164^

Best subset selection techniques have also been explored for dimensionality reduction, such as selecting the optimal subset of principal components. Similar to the above, “[*t*]*he first few PCs will only be useful for discriminating between groups if within- and between-group variation have the same dominant directions. If this does not happen* (*and in general there is no particular reason for it to do so*)*, then omitting the low-variance PCs may actually throw away most of the information in x (input) concerning between-group variation*”*.* ^165^ Other, albeit more computationally demanding selection methods have been used in areas such as spectroscopy and were discussed in the section on feature engineering (e.g., genetic algorithms). The use and potential benefits of subset selection for established fast voltammetry techniques, such as PCR, require further investigation. Regardless, the best subset selection adds additional computational time on top of dimensionality reduction or regularization approaches.

### 5.3 In Vivo Data for Model Training and Selection

In the absence of quantitative in vivo validation, qualitative in vivo validation is still an option. For example, Movassaghi and co-workers showed that the number of components selected by cross-validation did not track with multiple in vivo validation challenges when using a multielectrode training set.^26^ Instead, a subset of components yielded a worse cross-validation score but showed an improvement in in vivo domain knowledge results. As noted by the authors, in vitro validation may not translate to in vivo; thus, the selection of components by cross-validation may be suboptimal. Earlier reports also employed in vivo validation checks (e.g., comparing unexpected food rewards at the beginning and end of a recording session as a proxy for valid in vivo dopamine training sets),^5,166,167^ and model selection can be performed using these data as validation.

These qualitative model output checks contrast with other validation techniques that select components or reject test data based on maximal signal reconstruction (e.g., variance threshold, F-test, and Q_α_). It may be more helpful to frame in vitro to in vivo translation not as an issue of signal reconstruction but as a subset selection problem. Forcing the selection of a model based on its ability to reconstruct a signal in one domain does not guarantee its generalizability to another domain; this is especially true when multiple electrodes are involved. Thus, optimally selecting variables or components may also require considering the validity of built-in in vivo qualitative checks. For example, component subsets can be chosen so that a known stereotaxic, pharmacological, or stimulation-based neurotransmitter release is qualitatively observed. In that case, applying them to unknown biological dynamic situations might be more reliable.

Another approach is to select hyperparameters or models using domain knowledge from in vivo experiments. For example, the predicted concentrations of test data can be compared across models with different numbers of components or training sets to determine which results align most closely with known biological ground truths (i.e., non-negative concentrations, stimulation-matched responses, and expected responses in genetically modified animals). To increase confidence, voltammetry experiments are often performed in brain regions with narrower neurochemical diversity, thereby ruling out certain interferents or off-target effects. Validating across different techniques (e.g., microdialysis) can engender further confidence in model predictions.^168–170^

Rather than using in vivo data to assess the validity of *in vitro*-trained models, an ostensibly more straightforward solution is to train on in vivo data. Nonetheless, in vivo training presents unique challenges. Movement artifacts occur when electrodes are implanted into awake, behaving animals. Impedance differences also occur during vs after implantation due to the foreign-body response and biofouling. Importantly, there is the issue of ground truth, which refers to the inability to accurately determine the true concentrations of analytes for in vivo training data. Metabolites, peptides, proteins, and ions, as well as various neurotransmitters, can confound model predictions. Different approaches to in vivo training have been studied and advocated. However, technical difficulties remain, especially for chronic electrode implantation.^5^ Nonetheless, approaches to monitoring biofouling or otherwise correcting for electrode sensitivity changes across implantation times have been developed.^136,171,172^

In vivo training sets can be compiled by recording voltammograms from brain regions with known responses to stimuli, such as dopamine and pH changes in the nucleus accumbens after electrical stimulation in the substantia nigra/ventral tegmental area (usually performed after collecting the in vivo experimental data (e.g., behavioral) to account for electrode fouling effects).^62^ The electrode is excised, and a calibration factor (e.g., in units of nA/μM) is generated using a traditional in vitro flow cell experiment. The in vivo voltammograms are then scaled by this sensitivity factor to estimate concentrations for the in vivo training set. Some groups maintain that in vivo training sets can only be used for one animal, at one brain location, and one electrode to prevent erroneous conclusions resulting from variations in calibration factors and voltammogram shapes (e.g., the peak oxidation potential of dopamine can shift by >100 mV between subjects^50^). The so-called unrepresentative training sets can lead to poor concentration estimates, especially at low signal-to-noise ratios.^50^ Meanwhile, others advocate for the use of “standard” or “universal” training sets across animals and electrodes. These training sets are “intersubject”, in that a training set on one animal and electrode is used to predict data from a different animal and electrode. The advent of this approach came about as chronically, as opposed to acutely, implanted electrodes were introduced.^173,174^

The advantage of acute recordings is that postfouled calibration factors obtained in vitro are likely to be relevant over the time scale of the in vivo experiment due to limited immune responses and electrode fouling. Chronically implanted electrodes are exposed to more dynamic environments over extended periods of implantation. Also, chronic recording electrodes are rarely implanted with stimulating electrodes, precluding the use of in vivo training sets obtained at the same location in the same animal. Alternatives include unexpected sucrose delivery for in vivo training data to produce dopamine transients. However, this approach can underestimate predictions relative to electrical stimulation training sets, as the increase in dopamine concentrations for sucrose reward^61^ is smaller than for electrical stimulation. Sucrose delivery is also less time-locked than electrical stimulation. More commonly, the “standard training set”, which uses electrical stimulation from separate subjects, is used. Extending in vivo training sets to neurotransmitters beyond dopamine is challenging because less is known about the effects of behavioral stimulation and which brain region(s) to target to obtain reliable single-neurotransmitter transients.

The use of intra- vs intersubject training sets for chronic and acute electrodes for dopamine recordings was reviewed extensively by Rodeberg et al.^5^ When between-subjects in vivo training data from different electrodes and animals were used to make predictions, dopamine transients were underestimated by up to 50% compared to within-subject predictions.^61^ This was attributed to the variance in voltammetry responses between animals and electrodes. Using a cross-subject/electrode training set also yielded different Q_α_ validation results, indicating that the validity of the training data, as defined by residuals, depends on whether within- or between-subject training sets are used.

Similar findings exist for multielectrode studies. As the number of electrodes and training set size increases, more data variance is introduced that is not directly attributable to dopamine or pH. Thus, principal components bear less resemblance to their analytes and can cause overfitting. Again, PLSR and regularization may avoid this issue by their ability to separate contributions solely from variance in the data that covary with concentration or by avoiding dimensionality reduction entirely and instead penalizing each point in the voltammogram in a supervised manner. This could explain why multielectrode models have been successful outside of PCR.

Regardless, none of the aforementioned approaches are perfect; each has its downsides. While the work on within-subjects and electrode in vivo training has been developed into a robust procedure, it suffers from problems of scale. The data sets are inherently small. Further, all approaches rely on imperfect in vitro calibration to some extent. In vivo training sets are not guaranteed to be unconfounded (for example, H_2_O_2_ and adenosine can confound pH responses after electrical stimulation).^27^ They are also more time-consuming and cannot readily scale to multianalyte mixtures.

For these reasons, the use of in vitro training sets on postfouled electrodes to analyze in vivo data is gaining popularity, alongside alternative models to PCR. This in vitro approach enables the compilation of larger sets of training and test data, avoids additional in vivo experimentation, and can scale to any analyte concentration or mixture of analytes. Novel approaches that incorporate in vivo data into the training process are being developed in lieu of traditional template-based in vivo training sets. As transfer learning continues to mature, this will surely be an active area contributing new approaches.

Part of the reason for the growth in the popularity of in vitro training is the inability to carry out in vivo training for human voltammetry. To avoid calibration and electrical stimulation, human research often requires foregoing precalibration due to the potential contamination of sterilized electrodes. Work in humans, while still a small portion of voltammetry research, has contributed to the rise in the popularity of larger, more diverse in vitro training sets aimed at better generalization.

In summary, using semisupervised (transfer) learning represents a promising approach to addressing the generalizability issue by combining unsupervised and supervised methods. Geoffrey Hinton, one of the founders of modern machine learning, summarizes the potential power of this approach as follows. “*When we’re learning to see, nobody’s telling us what the right answers are*—*we just look. Every so often, your mother says ‘that’s a dog”, but that is very little information. You’d be lucky if you got a few bits of information*—*even one bit per second*—*that way. The brain’s visual system requires 10* ^*14*^ *[neural] connections. And you only live for 10* ^*9*^ *seconds. So it is no use learning one bit per second. You need more like 10* ^*5*^ *bits per second. And there’s only one place you can get that much information*—*from the input itself.*”^175^

## 6 Interpretability

The importance of model interpretability in scientific machine learning is gaining increasing attention.^176,177^ Interpretability provides context for understanding machine learning predictions and an avenue to improve model performance.

For component-based models such as PCR, visual inspection of the scores and loadings/regression vectors is essential and can be qualitatively assessed for chemical relevance.^50,70,160^ Hermans et al. visualized how different component selection procedures removed noise from dopamine and pH training sets. Keithley and Wightman interpreted the electrochemical behavior of regression vectors as a diagnostic tool for model evaluation. These authors incorporated domain knowledge regarding the chemical information contributed by additional components (e.g., the C, QH, and Q peaks in response to a pH change) in voltammograms.^50^ Interpreting the residuals can help to identify how training sets should be augmented as the peak potentials and sources of error can be pinpointed. Domain experts can then infer potential interferents that should be included in future training.^50^

Sparse, linear models help interpretability. Some papers have analyzed model coefficients to identify voltammogram subregions of importance. This includes methods such as PLSR (via loadings and VIP scores)^26^ and EN (via regression coefficients).^88^ Deep learning methods for interpretability have also been used. For example, Choi et al. looked at class activation feature maps.^36^ Mena and Buchanon et al. utilized feature importances.^130,131^ Xue et al. noted the features retained between single and mixed analyte solutions,^127^ and Twomey et al. examined embeddings.^86^

Background-inclusive voltammograms obscure analyte-specific features, which was a key reason for utilizing background subtraction (i.e., increased signal-to-noise and analyte peak oxidation and reduction feature identification). Machine learning has repeatedly been shown to use what was previously considered uninformative regions of voltammograms, even for applications outside of fast voltammetry, such as mechanistic electrochemistry.^178^ One of the most significant effects machine learning has had across voltammetry is to highlight the importance of nonfaradaic currents and motivate the use of new, information-rich waveforms.^4^

One of the largest effects machine learning has had across voltammetry is to question the importance of non-faradaic currents and motivate the use of new, information-rich waveforms.

## 7 Progress, Challenges, and Future Directions

Historical problems in voltammetry are converging toward machine learning-inspired solutions. The out-of-electrode and out-of-concentration prediction problems are improved with large training sets and appropriately trained models as well as subset selection methods, including novel statistical learning adaptations. The multiplexing issue is being overcome as models approach complex training sets with nearly double-digit numbers of analytes (Table 1). Rigorous validation will become even more crucial as techniques attempt to parse analytes with heavily overlapping signals (e.g., norepinephrine and epinephrine). With increased computational power and laboratory automation, more comprehensive training sets can be expected. However, the utility of larger networks and training sizes remains ambiguous. Still, the trend of head-to-head comparative studies should continue as there is no “free lunch” when it comes to model selection (i.e., no single optimization algorithm can be known a priori to outperform all others in all possible cases; a variety of models should be evaluated on a case-by-case basis).^38,179^

Some issues persist, such as out-of-distribution shifts. The utility of in vivo training sets has decreased, compared to in vitro training sets, in the context of domain knowledge validation. However, the latest advances in using transfer learning and other task-aware approaches may provide a compromise in utilizing both in vitro and in vivo training data. New developments in fast voltammetry, outside of machine learning, will also inform the appropriate solutions. Improved waveform design and electrode coatings and materials (both for working and reference electrodes), as well as a deeper fundamental understanding of biofouling at these interfaces,^162^ can be combined with the data analysis pipelines detailed here to address the remaining flaws after modeling. For example, the design of waveforms with a priori selectivity against interferents has been reported.^152^ Reproducibility of electrode construction and surface chemistries would help generalizability, such as standardized microfabrication.^24^ Still, this does not mean that voltammetry and machine learning should not be practiced in their current forms. These problems are pervasive throughout many bioanalytical realms, and new biology can and continues to be learned, even when using less-than-ideal techniques. Only through continued use will practitioners uncover and inspire new solutions to the challenges discussed here.

Successful models must be shared and made available for wide use. Models have little impact if others cannot implement and evaluate them. While we have reviewed state-of-the-art applications of machine learning to fast voltammetry, the most popular technique among neuroscientists remains the FSCV-PCR method, which was first implemented in 2004. As new models are developed, they need to be widely disseminated. The rise in the use of open-source machine learning packages is a significant step in this direction, moving away from previous custom laboratories or commercialized and licensed software. The most popular software is trending toward open-source packages written in R (glmnet) and Python (scikit-learn, TensorFlow, Keras, PyTorch), or commonly licensed academic software such as MATLAB. Developing tutorials or custom fast voltammetry packages that showcase these new techniques will help push the wider field beyond developers to utilize these techniques and discover new insights into the brain.^152,180^

The community could agree on an open-source baseline data set to compare metrics across models. This would be a great help.^181^ Large-scale publicly available databases often do not contain voltammetry data.^93^ In addition, agreed-upon community standards would be beneficial as benchmarks, as seen in a recently proposed framework for reporting key performance metrics of FSCV carbon electrodes, which aim to vary carbon materials.^163^ A similar framework for varying machine learning algorithms could be beneficial. This would allow head-to-head comparisons across models, training sets, and research groups, placing new techniques on an even playing field.

Universal databases and paradigms could enable large-scale studies. Similar studies have been conducted for spectroscopy and voltammetric electronic tongues as well as other slow-scan voltammetry techniques. However, few, if any, such wide-scale studies have been undertaken for fast voltammetry applications. While advances in model architectures and computational times are constantly being made, trade-offs are essential to consider if methods are to be applied beyond the few research groups developing new, fast voltammetry machine learning analysis techniques. At what point do minuscule increases in accuracy cease to impact important neurobiological conclusions?

Lastly, we should stand on the shoulders of our peers. This includes related fields, such as the voltammetric electronic tongue and mechanistic electrochemistry fields as well as more disparate or broad fields, including fundamental statistics, chemometrics, and spectroscopy. Many of the issues and problems inherent in voltammetry can be overcome by inspiration from these fields. Machine learning will continue to develop alongside fast voltammetry. Practitioners and developers of new methods are increasingly motivated to utilize state-of-the-art machine learning approaches. While the use of these techniques to predict real-time neurochemical dynamics in brains has not been perfected, advances and contributions to neuroscience will continue to be made.

## ∥

Department of Computational Biomedicine, Cedars-Sinai Medical Center, Los Angeles, CA 90048
